# The associated evolution of raptorial foreleg and mantispid diversification during 200 million years

**DOI:** 10.1093/nsr/nwad278

**Published:** 2023-11-02

**Authors:** Dahang Lai, Peichao Chen, Shumin Li, Xianzhe Xiang, Haohong Ou, Nuoyao Kang, Jingtao Yang, Hong Pang, ChungKun Shih, Conrad C Labandeira, Dong Ren, Qiang Yang, Chaofan Shi

**Affiliations:** School of Earth Sciences and Engineering, Guangdong Provincial Key Lab of Geological Processes and Mineral Resources, Sun Yat-sen University, China; School of Life Sciences, Key Laboratory of Conservation and Application in Biodiversity of South China, Guangzhou University, China; School of Earth Sciences and Engineering, Guangdong Provincial Key Lab of Geological Processes and Mineral Resources, Sun Yat-sen University, China; School of Earth Sciences and Engineering, Guangdong Provincial Key Lab of Geological Processes and Mineral Resources, Sun Yat-sen University, China; School of Earth Sciences and Engineering, Guangdong Provincial Key Lab of Geological Processes and Mineral Resources, Sun Yat-sen University, China; School of Earth Sciences and Engineering, Guangdong Provincial Key Lab of Geological Processes and Mineral Resources, Sun Yat-sen University, China; School of Earth Sciences and Engineering, Guangdong Provincial Key Lab of Geological Processes and Mineral Resources, Sun Yat-sen University, China; School of Ecology, Sun Yat-sen University, China; College of Life Sciences, Capital Normal University, China; Department of Paleobiology, National Museum of Natural History, Smithsonian Institution, USA; Department of Paleobiology, National Museum of Natural History, Smithsonian Institution, USA; Department of Entomology, University of Maryland, College Park, USA; College of Life Sciences, Capital Normal University, China; School of Life Sciences, Key Laboratory of Conservation and Application in Biodiversity of South China, Guangzhou University, China; School of Earth Sciences and Engineering, Guangdong Provincial Key Lab of Geological Processes and Mineral Resources, Sun Yat-sen University, China

## Abstract

Mantispidae have developed multidimensional specializations of predation that are leveraged by trade-offs involving attack properties, which is revealed by interdisciplinary analyses of phylogeny, morphometrics, and mechanical modeling. The lineage diversification was stimulated by its raptorial foreleg evolution, and was influenced by the ecosystem of corresponding periods, involving biotic and physical factors.

Adaptive evolution of predatory structure grants carnivores selective advantages [1], which evokes questions regarding the mechanism of functional adaptation via morphological variation and their potential impact on lineage diversification. Raptorial foreleg, as a key predatory structure in insects, is typically characterized by elongate coxae and stout femora possessing spines, and has evolved independently in many lineages [2]. Among them, Mantispidae have an evolutionary history fluctuating over 200 million years, along with their raptorial forelegs having transformed dramatically [3], which provide a conceptual example to address such questions. Here we assembled a dataset ranging over the entire geological history of the family. By combining phylogeny, morphometrics, and mechanical modeling, we show multidimensional specializations of predation that are leveraged by trade-offs involving attack properties. Raptorial foreleg evolution stimulated lineage diversification at varied tempos. Their interacting mode was also influenced by the ecosystem of corresponding periods, involving biotic and physical factors.

**Morphological disparity.** The evolutionary trajectories of raptorial foreleg morphologies, reconstructed jointly by Bayesian phylogenetic analyses and morphometric analyses on forefemur outlines using elliptic Fourier analysis and geometric morphometrics, reveal stepwise transformation (Fig. 1A and B). Mesomantispinae, the oldest subfamily ranging from the Jurassic to the Early Cretaceous, cluster around the principal component's coordinate origin of PC1 and PC2, characterized by a stout femur with rows of short spines, presumably representing the primitive state of the foreleg condition. The foreleg morphospace has expanded to become more disparate along PC dimensions ever since, as represented by the divergences of Doratomantispinae in the Late Cretaceous and the four extant subfamilies during the Cenozoic. Doratomantispinae occupy a significantly large morphospace, distantly isolated from the others in both canonical variate analyses and principal component analysis of geometric morphometrics. This pattern could be accounted for by the extremely elongated forefemoral major spine in Doratomantispinae, which is variably positioned within the subfamily and discrepancy in the presence or absence of secondary spines. Ancestral state reconstruction verifies the unelongated spine as the ancestral type of the family. The elongated major spine evolved independently among the subfamilies, followed by subsequent position variation in subclades, which implies differentiated adaptation at the functional and behavioral levels (see Supplementary Data).

**Figure 1.**
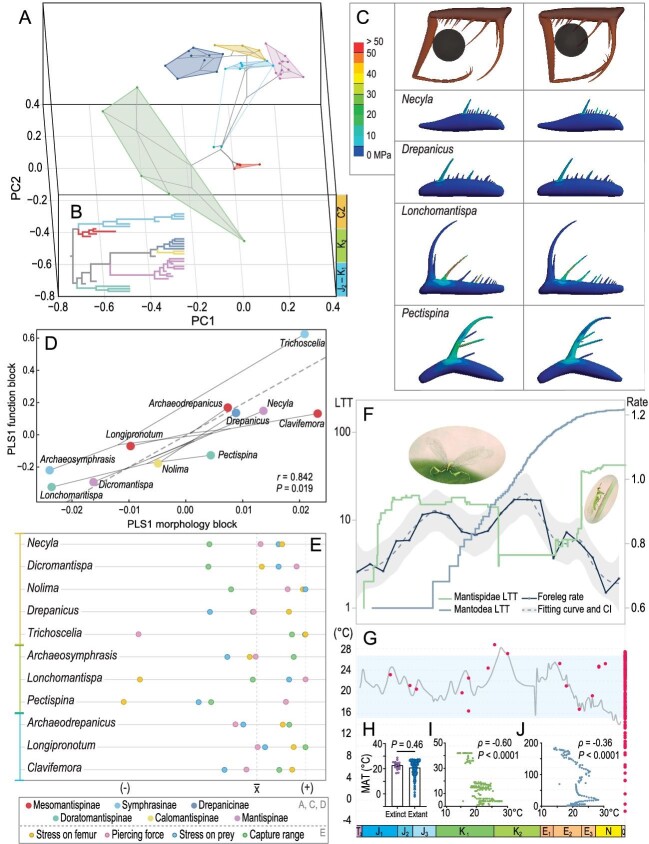
(A) Phylomorphospace of forefemur outlines over three phases of geological history. (B) Simplified phylogenetic tree of Mantispidae. (C) FEA of forelegs simulating clamping (left panel) and piercing (right panel) prey. (D) PLS analysis showing covariation between forefemur morphology and functional properties. (E) Functional indices mapped corresponding to positive (+) and negative (−) properties. All scaled to align the mean value of each index (x̄) and the maximum positive value, i.e. highest value of clamping force and capture range as well as lowest value of FEA stress and piercing force. (F) Foreleg rate weighted mean per 10-million-year bin, fitting curve and 95% confidence interval plotted on LTT of Mantispidae and Mantodea in logarithmic scale. (G) Habitat MAT of extinct and extant mantispids in corresponding time (red dots) mapped on global average temperature variation in last 200 million years. Blue shade indicates suitable MAT range. (H) Extinct and extant species habitat MAT as the mean ± s.e.m. and statistical test (n_Extinct_ = 16, n_Extant_ = 195). Spearman correlation between MAT and lineage number of (I) Mantispidae (n_Pairs_ = 205) and (J) Mantodea (n_Pairs_ = 148). Reconstructed artwork credit: Yuening Jian.

**Functional and behavioral adaptation.** The functionality of raptorial forelegs theoretically involves a series of physical properties including stresses on the leg, forces exerted on prey, and an effective capture range. We modeled forelegs across morphotypes and geological periods, then performed finite element analyses (FEAs). For comparing the morphological implications on functionality, the models were scaled to be equally long with only the outer layer, being of unique thickness, thus excluding the influence of internal structures and their physiological roles. Simulation of clamping simplified spherical prey items reveals a high differentiation of von Mises stresses on forefemoral spines as well as the entire forefemur among morphotypes (Fig. 1C). Most species show fine stress when clamping the prey. Stresses on morphotypes of short and median-long spines are not significantly different. However, forelegs with an extra-long spine display an exceptionally high stress on the forefemur and especially the most accessible spine. Comparison of FEA on penetrable versus impenetrable prey shows a significant decrease of stress on spines of Doratomantispinae whereas no evident difference is found in the basal and midway median-long spine morphotypes.

To estimate forces exerted on prey, mechanical experiments were performed on 3D printed models and simulated prey with either rigid, impenetrable surfaces or soft, penetrable surfaces (inflated). Results on impenetrable prey are not precisely in accordance with the morphotypes, although there appears to be a general trend in employing stronger forces toward the Recent (Fig. 1E). Experiments on penetrable prey show a strong force requirement for short spine morphotypes but generally a weak force requirement for long spine morphotypes. Morphotypes of extra-long spines required exceptionally small forces to impale their prey (see Supplementary Data). FEA and

mechanical experiments jointly demonstrate a functional divergence among the morphotypes. Forelegs with all short spines are adapted to clamp rigid prey with a strong force but are basically unable to pierce prey. Forelegs with median-long spines are capable of capturing both rigid and soft prey with strong force and have fine stress resistance. The extra-long spine morphotypes can easily pierce soft prey with less exertion and fine stress, but with high risk when capturing rigid prey.

Additionally, effective capture ranges were measured on scaled forelegs with equal femur length and femur-tibia angle. Rather than spine length, the range is more conditioned by the major spine position. It reveals effective capture ranges are generally at three levels, decreasing in line with the spine types: short spines, basal major spines, and midway major spines.

Synthesizing the aforementioned functionality indices, a corresponding divergent process to the PCA morphospace variation is evinced (Fig. 1A and E). Functional indices of mantispids in the earliest stage (J_2_–K_1_) are generally centralized around an average level, underpinning stability. They have a distant capture range and exhibit low stress when hunting, but less power of attack. Specialization of foreleg function later emerged during the early Late Cretaceous, represented by the Doratomantispinae exploiting new ecological opportunities. Their piercing specialized forelegs show divergence in effective capture range and applied force. The Cenozoic mantispids tend to be further specialized in disparate directions. Overall, they show better efficiency and improvement in certain abilities. Phylogenetic partial least square analysis reveals a significant and strong correlation between forefemur morphology and functional properties (Fig. 1D), suggesting that the adaptive evolution of predation functionality was attributable to their morphological modification.

**The adaptive radiation.** Evolutionary rates of foreleg changes reveal that positive selection mainly occurred during the subfamilial divergences, whereas stabilizing selection mostly occurred among intrasubfamilial genus-level branches. It suggests that foreleg morphological transformation is a causal factor for higher rank divergence while stabilization is a feature of lower rank diversification within a clade. The changes of significantly accelerated rates are mostly associated with major spine transformation, further demonstrating the great impact of the functional major spine variation to foreleg evolution as well as to lineage diversification (see Supplementary Data).

A lineage through time (LTT) plot of Mantispidae, generally in accordance with net diversification rate variation (see Supplementary Data), illustrates two phases of evolutionary radiation, but shows an unlike correlation between that and foreleg change rate (Fig. 1F). As the first phase, mantispids exhibited a rapid early accumulation of lineages concomitant with bursts of foreleg morphotypic evolution during the Jurassic. This joint acceleration is presumably attributed to effects of the preceding end-Triassic mass extinction, after which mantispids benefited from the vacant niches and ecological opportunities [4]. Along with the following terrestrial ecosystem recovery, mantispids diversified in concert with foreleg modification, consequently shaping the basic blueprint for the family.

The second radiation occurred from the Eocene through Holocene, when, intriguingly, lineage radiation lagged behind foreleg disparity. The forelegs achieved maximum disparity during the Late Cretaceous, then dramatically decreased during the Paleocene, and rebounded in the Eocene. The forelegs’ morphology diversified during the Angiosperm Terrestrial Revolution, plausibly fueled by the ascendancy of angiosperms and their corresponding phytophagous insects, which further provided partitioned niches and revolutionized ecospace [5]. Consequently, the functional differentiation in forelegs of extant morphotypes formed, achieving advanced disparate predatory strategies during the Cenozoic. The lineage number rose slowly during the Eocene and had a dramatic ascent during the Oligocene, eventually maintaining a plateau of maximum diversity to the present. This phenomenon suggests foreleg disparity triggered the mantispids radiation during this stage, which is commonly observed in other invertebrate clades [6].

The rise of mantises plausibly influenced the evolutionary pattern of mantispids during this phase. Mantodea LTT plots revealed a constant increase during the Cretaceous [7], thereafter turning into the currently most speciose insect group with raptorial forelegs (Fig. 1F). Due to ecospace overlap and niche competition, Mantispidae differentiated in and accommodated to their ecospace by increasing foreleg disparity prior to lineage diversification. Modern mantises have larger body size with the forefemoral major spine hinged and flexible, indicating detailed functional and behavioral deviation as well as ecological differentiation at micro scales [8].

In the physical environment, global temperature changed dramatically in the Late Cretaceous, reaching a maximum during the last 200 million years [9]. Species distribution modeling for extant mantispids found the mean annual temperature (MAT) contributes most to their distribution, with suitable habitat ranging from 15.3°C to 27.1°C. The noticeable absence of occurrences where the MAT exceeds 28°C indicates unfavorable conditions for them at higher temperatures. Tracing their habitat MAT back into geological time finds no significant difference between the extinct and extant data, suggesting the habitable temperature range did not change notably. A significant negative correlation is found between the mantispid LTT and global MAT, whereas the mantodean LTT is less related, which implies rising temperatures had a more severe effect on mantispids (Fig. 1G–J, Supplementary Data) [9,10]. Consequently, the Cenomanian–Turonian Thermal Maximum and the ensuing intense temperature fluctuation probably acted as another crucial cause in the resulting low lineage number of Mantispidae in the Late Cretaceous. They radiated afterwards when global temperature reverted back to their suitable range. Mantispidae present a spectacular case of predatory insect evolution driven by functional structure transformation, meanwhile influenced by the varying background environments.

## Supplementary Material

nwad278_Supplemental_Files
