# Supplementary material for: The associated evolution of raptorial foreleg and mantispid diversification during 200 million years
Source: Natl Sci Rev. 2023 Nov 2;10(12):nwad278. doi: 10.1093/nsr/nwad278 (PMC10686013; doi:10.1093/nsr/nwad278)
Supplement: nwad278_Supplemental_Files [file nwad278_supplemental_files.zip › Supplementary Data (Text, Figures & Tables).pdf]

SUPPLEMENTARY DATA

The associated evolution of raptorial foreleg and mantispid diversification during 200 million years

Dahang Lai†, Peichao Chen†, Shumin Li†, Xianzhe Xiang, Haohong Ou, Nuoyao Kang, Jingtao Yang, Hong Pang, ChungKun Shih, Conrad C. Labandeira*, Dong Ren*, Qiang Yang*, Chaofan Shi*

Contents:

Phylogenetic analyses

Phylogenetic relationships and time calibration

Ancestral state reconstruction

Evolutionary rate estimates

Lineage through time plot

Phylogenetic partial least squares

Morphometrics

3D modeling and Finite element analyses

Mechanical experiments

Effective capture range measurement

Behavioral and ecological exploitation during a geological window

Species distribution modeling and statistical tests

Supplementary Figures S1 to S27

Supplementary Tables S1 to S11

Supplementary Data list

References

Phylogenetic analyses

Phylogenetic relationships and time calibration

To test the phylogenetic relationships of Mantispidae along with their geological history and build a backbone for further analyses of foreleg morphological and functional evolution, Bayesian tip-dating analyses were performed. The data matrix consists of 106 morphological characters and 37 taxa updated from previous studies [1,2] (Supplementary Data 1). Ingroups include eighteen extinct taxa covering representatives from geological formations with fossil records of Mantispidae and fourteen extant genera involving all genera of Symphrasinae, Drepanicinae, Calomantispinae and selected genera of Mantispidae (Table S10). Most of the fossil records are genera except for two species of *Dicromantispa* considering distinct geological distribution. One extinct and one extant genera of Berothidae, along with one extinct and two extant genera of Rhachiberothidae, were selected as outgroups [3–7]. One extinct species, *Gerstaeckerella asiatica*, from the Late Cretaceous Kazakhstan, has been described and assigned to the extant genus *Gerstaeckerella* of Drepanicinae [8]. However, the holotype of the species has only the hind wing preserved with limited venation characters. The phylogenetic status of the species cannot be verified in the analysis. Furthermore, the topology of the phylogenetic tree became less resolved because of too many missing characters. Therefore, this species and a few other fossil taxa were excluded in the final analyses due to inadequate valid characters. Consequently, the divergence time, especially of Drepanicinae and their deeper nodes, needs further study with more and better-preserved fossil specimens in the future.

Recently, a few studies recovered controversial phylogenies within the superfamily Mantispoidea, along with discussion on their relationships based on morphological taxonomy. It principally involves different interpretations on the familial assignment of Symphrasinae, and their relationship with Rhachiberothidae, otherwise Rhachiberothinae, as a subfamily,

belonging to either Mantispidae or Berothidae [9–12]. We tentatively followed the traditional classification in this study, considering Symphrasinae as an ingroup of Mantispidae while Rhachiberothidae as an outgroup, for two reasons. First, phylogenetic analyses based on data with more extensive sampling of fossil taxa agreed with the traditional classification [1,13]. Second, clarification of the phylogeny of the superfamily needs further investigation based on a more comprehensive and extensive dataset, which exceeds the intent of this study.

The chronological constraints of fossil taxa, i.e., the lower and upper bounds of the geological ages, were used for Bayesian tip-dating analyses. The chronological constraints of fossil taxa referred to the database Lacewing Digital Library [14] (LDL, https://lacewing.tamu.edu) and original references. The matrix contains morphological characters from the head, thorax, abdomen, leg and wing. Characters were coded from the original literatures, photos, and examination of type specimens. All characters were treated as discrete and unordered. Taxa were coded as polymorphic when a taxon has more than one state, as "-" when the character is inapplicable, and as "?" when the character state is unknown. Characters were subdivided into three partitions for regional evolutionary rates estimates. Partition I includes 21 characters from the foreleg. Partition II includes 32 characters from the forewings. Partition III includes 53 characters from other body parts. Separate morphological clocks were assigned to each partition, in order to obtain separate rate estimate of character evolution for distinct regions.

The phylogenetic analyses were run in MrBayes 3.2.7 [15]. We performed tip-dating analyses to co-estimate the tree topology, divergence times, and evolutionary rates. The fossilized birth-death process (FBD) was used as a prior for the timetree [16,17]. The tree age prior for FBD process was assigned an offsetgamma prior with mean age of 178.4 Ma and minimal age of 174.1 Ma, derived from the oldest fossil mantispid *Liassochrysa* [14,18,19]. We used the stepping-stone sampling to estimate the marginal likelihoods of different model

combinations. Two distinct clock models were tested: the uncorrelated independent gamma rates (IGR) [20] and the autocorrelated Thorne–Kishino 2002 (TK02) models [21]. For the model of character rate variation, we tested models under gamma and lognormal distribution. The results of marginal model likelihoods for the assessed different model combinations are not significantly different, revealing highest value for the IGR clock model combined with gamma-distributed rate model (Table S1), which was used for the subsequent analyses.

We executed two simultaneous, completely independent runs in Markov chain Monte Carlo. Four chains (three heated chains and one cold chain with temperature 0.04) were used per run for 40 million generations and sampled every 2000 generations. The first 25% samples were discarded as burn-in. The remaining samples from the two runs were combined. The posterior trees were summarized as a 50% majority rule consensus tree (Fig. S2) and a maximum compatible consensus tree (Figs. S1, S7).

Supplementary results and discussions

The phylogeny reveals that the four extant and two extinct subfamilies are validated with a few fossil genera as stem to subclades. The time-calibrated tree suggests that the family originated in the Early Jurassic, a result that is congruent with molecular estimates [22]. Divergence time estimation reveals two main phases of Mantispidae radiation: (i) a phase from Early Jurassic to Early Cretaceous and (ii) a phase during the Paleogene (Figs. S1, S2, S6, Table S2).

<u>Ancestral state reconstruction</u>

Ancestral state of foreleg characters was estimated using MrBayes Ancestral States with R (MBASR) based on the time-calibrated phylogeny using partitioned morphological clocks [23]. The characters and their corresponding states are listed in Table S3. The reconstruction results are shown in Fig. S7A, Figs. S8–S13. In terms of the states of character

I (forefemoral major spine), we define “absent” as all femoral spines of very close length and significantly shorter than the width of forefemur; “long spine” as the spine significantly longer than most other spines and as long as the width of forefemur; “extra-long spine” as the spine significantly longer than the width of forefemur and as long as the length of foretibia. The thornlike structures on the forefemur in Mantispidae, we termed “spine” here, are typically formed by a cuticular extension bearing modified seta at the end, varying on the ratio of the cuticular extension length to the seta length among taxa. This structure is also termed as “integumentary process (IP)” in ref [24].

Supplementary results and discussions

Ancestral state reconstruction of forefemoral major spines suggests that the extra-long spine likely originated during the Late Jurassic primarily in a basal position, and later located in the midway along the forefemora. The median long spine likely originated independently during the Cretaceous and Neogene, and its position subsequently varied. The remarkable foreleg disparity in concert with the repeated development of the major spine through geological history implies differentiated adaptation at the functional and behavioral levels.

Evolutionary rate estimates

Evolutionary rates were estimated for integrated morphological characters and partitioned characters of foreleg, forewing, and other body parts respectively. The 106 characters in the matrix were first treated as a single partition for the overall evolutionary rate estimates (Fig. S3A). The characters were next subdivided into three partitions and a separate morphological clock was assigned to each partition for estimating the evolutionary rate of each partition (Figs. S3B, S4A, S5A). Relative rates of separate region evolution were calculated for representing selection strength variation among branches during the geological history using partitioned morphological clocks following ref [25], which was indicated by the rate

scalar ratio [26] and adopting the threshold for distinguishing positive and stabilizing selection of ref [15] (Figs. S7B, S4B, S5B). The median value of foreleg rate on each branch was used to calculate and plot the rate curve. The geological span of Mantispidae were divided into 10-million-year time bins. Branches falling into each bin and the respective branch length within the bin were collected for the next statistical analyses. The weighted mean rate of each bin was calculated according to branch lengths within the bin. A fitting curve and 95% confidence interval were plotted using LOESS regression (Fig. 1F). The calculation and plotting were performed in R 4.1.0 (R core Team).

The fossil record of Mantispidae shows a distinct distribution among the Mesozoic periods and epochs (Table S10). For estimating the net diversification, relative extinction and relative fossilization through time, the geological history with fossil record of mantispids was subdivided into four time bins by the boundary ages: 145 Ma, 100 Ma, 66 Ma. The macroevolutionary parameters were estimated independently for the Jurassic (Origin–145 Ma), the Early Cretaceous (145–100 Ma), the Late Cretaceous (100–66 Ma) and the Cenozoic (66–0 Ma), using MrBayes 3.2.7 [15] and Tracer v1.7.2 [27]. Summary statistics are shown in Fig. S6 and Table S2.

Supplementary results and discussions

The analyses with the integrated and partitioned characters matrices find similar results agreeing on all the subfamilies monophyletic but different in their relationships, mainly involving the phylogenetic status of Doratomantispinae. We applied the result from the integrated matrix in most of the work and used the result from the partitioned matrix when referring to the foreleg evolutionary rates.

The foreleg evolutionary rates show three acceleration phases, i.e., during the Middle Jurassic to the Early Cretaceous, the Late Cretaceous and the Paleogene, with the Late Cretaceous highest (Fig. 1F). This result generally corresponds to the chronological

morphospace recovered in the EFA (Fig. 1A). The morphospace expanded significantly in the Cretaceous and slightly contracted in the Cenozoic, especially within subfamilies. An analogous pattern was also found in ref [28], although with certain differences on the change extent because of the period and group division and taxa selection in the analyses.

Lineage through time plot

Lineage through time (LTT) plots of Mantodea and Mantispidae were performed for comparing the diversification histories of the two largest insect groups bearing raptorial forelegs. LTT plot of Mantodea was based on the time-calibrated phylogeny of ref [29] combined with the order node dating of Mantodea in ref [30] (Figs. 1F, S14B). The tree used in LTT plot retained all nodes provided with estimated divergence times in the reference. LTT plot was obtained utilizing function 'ltt' of the R package *phytools* v1.0-3 [31,32]. There are 185 retaining terminal tips, proximal to the number of sampled genera in the analysis (179/436 genera (41%)) [29]. In a later work on the higher rank phylogeny of Dictyoptera, the node date of Mantodea was updated and extended earlier [30]. Therefore, we adopted this updated root date combined with the internal node dates from preceding analysis in the plot. LTT plot of Mantispidae was based on the time-calibrated phylogeny using a single morphological clock partition. For a more comprehensive coding in detailed morphological characters, the taxa in the phylogenetic analyses were selected, but this may bias the LTT plot. Therefore, we added all the published extinct and extant genera of the family to the tree, attaching to the node according to taxonomic assignment (Figs. 1F, S14A). Considering differences in taxonomic ranks of the two groups, it is difficult to compare the diversification history of the two under an appropriate criterion. It should be clarified that the LTT plots were mainly based on generic level in both groups. But it was not fully sampled in Mantodea. The number of terminal tips is about 42% of recorded genera and 3 times of tribes. The two LTT plots were solely aiming at

a comparison of the diversification variation through the geological history.

Supplementary results and discussions

LTT plot of Mantispidae illustrates two phases of evolutionary radiation. The resulting pattern generally is in accordance with net diversification rate variation calculated by a Bayesian phylogenetic tip-dating analysis (Fig. S6, Table S2), although the net diversification values were estimated from partial sampled data. LTT plot show highest lineage number in the Cenozoic while net diversification rate of the Jurassic is higher than that of the Cenozoic. Moreover, the lineage number in the Early Cretaceous is slightly lower than that in the Jurassic, but the net diversification rate in the Early Cretaceous is only half of that in the Jurassic. Both results agreed that the lowest diversification was in the Late Cretaceous.

The evolutionary pathways of mantises and mantispids differ from trajectories taken by most other hemimetabolous and holometabolous lineages, i.e., the former diversified earlier than the latter [33]. This is plausible due to the early origin of Neuroptera, including Mantispidae, among the Holometabola [9,30,34,35].

Phylogenetic partial least squares

The phylomorphospace and functional analyses of forelegs show great variation and a specific pattern through the evolutionary history of Mantispidae. To test their covariation on a phylogenetic basis, phylogenetic partial least square (PLS) analysis was performed based on blocks of morphology and functional properties. Eleven genera, of which forelegs were modeled and tested for functional properties in this study, were used in the PLS analysis. The phylogeny of the eleven taxa followed the phylogenetic tree using a single morphological clock partition. The morphological block contains the first five PCs of EFA, describing over 80% of the outline variation (83.77%). The functional block consists of the four indices of functional properties. PLS was analyzed using function 'phylo.integration' of the R package *geomorph*

v4.0.4 [36,37].

Morphometrics

The forefemora exhibited the highest diversity in Mantispidae. To quantify the morphological disparity among forefemora, morphometrics of forefemur outlines were analyzed based on a dataset illustrating variation across all geological periods and subfamilies of Mantispidae, including 27 extant and 16 extinct, totally 43 species. In elliptic Fourier analysis (EFA), the shape variation of forefemoral outlines was quantitatively evaluated based on Elliptic Fourier Descriptors (EFDs) by SHAPE v1.3, as well as principal component analysis (PCA) of the coefficients of the EFDs, using 40 harmonics [38,39]. Phylomorphospace was plotted using function 'phylomorphospace' of the R package phytools v1.0-3 (Fig. S15A) [32,40]. Geometric morphometrics (GM) is mainly based on the Cartesian coordinates of landmarks, commonly using Generalized Procrustes analysis (GPA) [41]. We adopted both methods to analyze on the same dataset for verifying the results. We used 400 curve semilandmarks to delineate the forefemoral outline. GPA was performed on all the semilandmarks to superimpose them to a common coordinate system by rotating, translating and scaling the landmarks using function 'gpagen' of the R package *geomorph* v4.0.0 [42,43]. PCA was performed for interpreting the femoral outline variation by function 'gm.prcomp' in R package *geomorph* (Fig. S16A). Canonical variates analyses were performed by function 'CVA' in R package *Morpho* v2.9 using the first 20 PCs of EFA and GM respectively [44,45] (Figs. S15B, S16B).

Supplementary results and discussions

The results revealed a concurrence (Figs. S15A, S16A). In both analyses, the first five PCs described over 80% of the outline variation. The first two PCs explained the majority of variation in the dataset. In EFA, PC1 accounts for 35.26%, PC2 accounts for 19.56%. In GM,

PC1 accounts for 46.39%, PC2 accounts for 21.77%. All other PCs account for less than 15% in both analyses. The phylogeny used in phylomorphospace was constructed based on the backbone tree from single morphological clock partition. Results of Canonical variates analyses show similar patterns to morphospaces of the respective first two PCs, but more tightly clustered within subfamily and more distinguished among subfamilies, especially for Doratomantispinae from the others (Figs. S15B, S16B).

Both EFA and GM provided morphospace polygons, illustrating forefemur morphological disparity among subfamilies, though with some overlap (Figs. 1A, S15, S16). EFA reveals a large overlap between Mesomantispinae and Symphrasinae. Subfamilies are principally distinguished along the PC1 axis, with the exception of Calomantispinae that is distinct from the cluster of Mesomantispinae and Symphrasinae along the PC2 axis. The results show the oldest subfamily Mesomantispinae (J-K_1) cluster around the principal component's coordinate origin of PC1 and PC2. The foreleg morphospace expanded to more disparate PC dimensions in the Late Cretaceous, represented by the divergences of Doratomantispinae, and subsequently the four extant subfamilies during the Cenozoic. The major spine is more prevalent in extant mantispids, but of median length and varies in position.

3D modeling and Finite element analyses

Forelegs of extinct and extant representatives were 3D modeled referring to measurements of extant specimen. The three-dimensional models were scaled to an equal forefemur length of 3 mm, the mean length of sampled forefemora (Table S4). The outer layer of models was 0.02 mm in thickness, referring to measurement on computed tomography images of the forefemur cuticle of *Necyla* (Supplementary Data 2). Foreleg of *Necyla* was scanned by high resolution X-ray computed tomography (nano-Voxel-3000). Segmentation and reconstruction of 3D models were processed in Avizo 2019.1. Cuticle thicknesses were

measured on the CT slices in Ansys SpaceClaim 2021 R1. Mean value of thicknesses from different spots was used for 3D modelling and analyses subsequently. Models were treated as homogeneous and isotropic. The elastic modulus (E) of *Necyla*'s forefemur was measured by atomic force microscopy and used for subsequent analyses (E = 3600 MPa). We performed finite element analyses (FEA) on models that were first simplified along isolated spines with gradient loadings and later based on models simulating capture performance.

Eleven species were selected representing the main morphotypes recovered from morphometrics and principal geological intervals. In the capture process, mantispids mainly use forefemur, foretibia and foretarsus to strike, trap, fix or kill the prey. Therefore, we 3D modeled these three elements for each species in Blender. Models were treated as homogeneous and isotropic. The mechanical properties of the foreleg would have been affected by their morphological and physiological properties. In this study, we are focusing on the implications of morphology transformation. So only the cuticular out layer of forelegs were modeled in the analyses, though other aspects should also influence the mechanical properties, such as the non-uniform of the cuticle and their internal structures and properties, i.e., muscle, resilin, etc. [46]. The 3D models were further processed and converted to NURBS models in Geomagic Studio 2013 and Ansys SpaceClaim for finite element analyses (FEA) and printed out for mechanical experiments (Supplementary Data 3–13).

Elastic modulus of foreleg is an important parameter of material property, indicating the ability of the cuticle to resist elastic deformation. We measured Young's modulus of foreleg of *Necyla* by atomic force microscopy (AFM) at ten different spots over the forefemur surface and used the mean value for subsequent finite element analyses. Force curves were obtained by the experiment and the elastic modulus was calculated according to the Hertz model by the following equation [47]:

$$F=\frac{4}{3(1-\upsilon^2)}ER^{1/2}\delta^{3/2}$$

Finite element analyses were implemented in Ansys Workbench (v. 2021 R1) using solid mesh FE models. Each model consists of approximately 100,000–150,000 four-node tetrahedral elements. The modeled layers were equally thick of 0.02 mm, measured from CT images. The analyses were constrained to rigid body motion at the contact spots. FEA were performed in different scenarios to test the stress on forefemur when hunting. The simulated prey was simplified to be spherical. The simulated prey size was pre-tested with diameter of 0.8, 1.0, 1.2 mm. A consistent diameter of 1.2 mm was applied in the following FEA analyses due to it is the size of which the prey could be stably held by forelegs of all morphotypes with certain numbers of spines contacting the prey. Although in reality, the mantispids are not necessary to prey on the most appropriate size, we assume for comparing the functional discrepancy, it is reasonable to use the appropriate size. Firstly, forces were applied along isolated femoral spine from $1x10^{-3}$ N to $3x10^{-3}$ N with a gradient of $5x10^{-4}$ N. Secondly, simulation of foreleg clamping impenetrable prey was performed. Forces were applied perpendicular to tibia with loading of $6x10^{-3}$ N. Thirdly, simulation of foreleg piercing penetrable prey of the same form, size and material was performed with the same loading as the second set of analyses. Results of analyses along isolated femoral spines are shown in Figs. S17–S21. Results of simulating clamping impenetrable prey are shown in Fig. S22. The mean value of von Mises stress on the entire forefemur of each model was used as an index in PLS analysis (Table S5). Results of simulating piercing prey are shown in Fig. S23.

Supplementary results and discussions

(1) The result indicates that the foreleg with an extra-long spine is very vulnerable and easily fractured when clamping prey, which likely leads the specialization of the Late Cretaceous Doratomantispinae toward a novel adaptation. Instead of a hypothetical prey with rigid surface, we alternatively used a hypothetical prey with soft and penetrable surface to conduct the FEA, which provided a clue to the adaptation for predation in Doratomantispinae.

(2) *Pectispina* are unique for their major spine pectinate and very few other spines directly on the femur. Compared to the other Doratomantispinae genus in the analyses, *Lonchomantispa*, *Pectispina* show better equilibrium on stress among spines in most analyses, indicating relatively less risk for individual spine (Figs. S17–S23). On the other hand, the genus show less advantageous than *Lonchomantispa* in other aspects of physical properties (Fig. 1B).

(3) The FEA analyses also show a heterogeneous distribution of stresses on spines of most morphotypes, especially in the long and extra-long spine morphotypes. The distal face of the spine generally undertakes a higher stress than the proximal face when clamping rigid surfaced prey, especially around the spine base (Figs. 1B, S17–S22). This is congruent with the material composition of the spine, that the proximal face is resilin-dominated while the distal face of the spine is strongly sclerotized [48], which is supposed to offer the capability of undertaking higher stresses.

Mechanical experiments

The 3D printed models were used for mechanical experiments. Both forelegs and hollow cylinders simulating rigid prey were printed using photosensitive resins of highest elastic modulus (E = 2370–2650 MPa, μ = 0.41) to simulate arthropod cuticle. The printed forelegs were scaled to the same length of femora as 100 mm. The models were magnified for better illustrating the detailed structure of the forelegs due to limited printing resolution, consequently resulting in differing orders of magnitude in FEA and mechanical experiment results. But the results should represent the comparison among foreleg morphotypes within each experiment or analysis set. Cylinders are 30 mm in diameter and 50 mm in length. Soft surfaced prey was simulated by penetrable air sac made of polyethylene. The fully inflated air sac is 20 mm in diameter, 200 mm in length and 45μm in thickness. The simulated prey was tested with gradient diameters of 20, 30, 40, 50 mm, and the size appliable to all foreleg models

in either scenario was applied in the subsequent experiments.

The strain and stress of the contact spots on cylinder were measured by TST3827E dynamic data acquisition system with attached strain gauges when the cylinder was clamped by the leg model. For each measurement, a consistent force of 3 N was applied perpendicular to the tibia by a dynameter, and all the contact spots by spines of the testing foreleg were measured.

In the clamping experiments, stress data were obtained by dynamic data acquisition system for each contact spot by forefemoral spine. The stress curves show stress variation of the contact spots along time. The stress difference between the stable state when force is applied and the state when force is removed represents the stress on the spot generated by spine. And the greatest stress difference represents the greatest stress a prey endured from the foreleg clamping. For each foreleg model, we repeated the experiments to confirm concordance of result curves and used result curves of one experiment to process the data. In each experiment, we chose the curve of greatest stress difference, representing the spot which endured greatest stress in the experiment. The last five to ten seconds of force applied stable state and the first five to ten seconds of force removed stable state of the curve were extracted. And the median value of stress in each extracted time bin was calculated. Then the difference between them was used as the stress generated by this foreleg model in the simulation experiment (Fig. S24A). The extracted time bins and respective data were shown in Fig. S24B–L. The values used in the PLS analysis and Fig. 1E were as in Table S6.

In experiments simulating impalement of soft surfaced prey, a tibia was loaded by a dynameter when the fully inflated air sac was placed exactly between forefemur and foretibia. The force required to impale the air sac was recorded. When the air sac was stably placed between forefemur and foretibia, it would be impaled by the most accessible spine, which, in many cases, is not the major spine. Experiments were also executed to test the force

requirement for the major spine to impale the air sac by purposely placing the air sac at a special position. Experiments of each scenario were repeated for three times and the mean value was obtained. The results unexpectedly show the major spine required greater force than the most accessible spine to impale the simulated prey. It indicates that the foreleg with major spine was advantageous to impale prey, but most possibly not impale using the major spine. While the major spine plausibly acted to trap the prey from escaping. The mean values from the experiments of impalement by most accessible spine were used in the PLS analysis and Fig. 1E, for this should be the more practical and reasonable scenario. The results of experiments simulating impaling prey are shown in Table S7.

Effective capture range measurement

The elongate foreleg elements along with prothorax extend the possible hunting distance for mantispids. They adopt a jack-knife mechanism for preying, jointly accomplished by forefemur, foretibia and foretarsus. As a result, the lengths of these elements affect the range in which the mantispid is likely to strike and capture a prey item. The distance from tibia/tarsus tips to femur base should have been the range for a mantispid to capture prey, if not considering the impact of spines. However, many mantispids developed a long major spine on the basal or midway of forefemur. Different from the hinged major spine on mantises forefemora, the major spine on mantispids is rigid [49]. So, the presence of a rigid major spine would narrow the capture range to certain extent because only the distance from tibia/tarsus tips to the femoral major spine tip is effective for the mantispid to strike and capture a prey item [1]. However, the major spine may have advantages to the mantispid in other aspects (see FEA and mechanical experiments). Consequently, the capture range is considered as an index of the functional properties. The ratio of the measured range to femur length at criterial angle was calculated and used in the PLS analysis and Fig. 1E. The results are shown in Table S8.

Effective capture ranges were measured on the foreleg models. In view of different curvatures among the sampled forefemora and foretibiae, the line connected the proximalmost and distalmost points of each leg element represents the direction of the element. Forelegs were firstly placed with the forefemur direction horizontal and the femur-tibia joint open till the dorsal surfaces adjacent to the joint of both elements parallel. The angle between directions of the two elements was measured. The minimum angle of all samples, 140°, was taken as the criterial posture for measuring capture range of all samples. Length from tibial distal to femoral major spine tip for major spine present legs or femoral proximal for major spine absent legs was measured. Indeed, the effective capture range should be from major spine tip or femoral proximal to pretarsal claws. But a few fossil taxa have the tarsi and claws unpreserved. For comparison under a consistent criterion, the range was measured to tibial distal.

Behavioral and ecological exploitation during a geological window

Among the foreleg morphotypes of mantispids, Doratomantispinae are peculiar in morphospace occupation and functional properties. Considering their extraordinary and delicately constructed femoral spines, their hunting behavior and type of sought prey posited herein indicates a unique fitness mode. Small-sized arthropods, especially insects, have been the common and obtainable prey for modern mantispids [48]. However, the exoskeleton of arthropods was not easy to penetrate for Doratomantispinae. Armed with an extra-long spine, which was more advantageous for piercing than for clamping, Doratomantispinae would have preferred to consume soft skinned prey. In such a circumstance, a vertebrate of appropriate size, for example, a small, juvenile reptile, or an insect larva, could be a preferable prey option for Doratomantispinae [50–52]. In contrast with a rigid exoskeleton, the relative soft skin of vertebrates would reduce energetic expenditure for Doratomantispinae. As a cost, the raptorial

femoral weapon was vulnerable for clamping rigid prey, such as more widespread arthropods, which indeed provided more calories compared to reptiles of the same fresh mass [53–55]. Nevertheless, the differentiation of prey categories would have facilitated Doratomantispinae to exploit new ecological niches. The diversity of Doratomantispinae species further suggests that the selective advantages of an extra-long spine associated with its piercing behavior benefited this lineage to certain extent for their prevailing in the Burmese community of the Late Cretaceous [1,2,13,56]. Unfortunately, the piercing mechanism of the specialized foreleg seems to be a less than optimum adaptation by assuming an extra risk and resulting in restricted prey options. This limitation could explain their short geochronological lifespan as well as their geographical rarity. Late Cretaceous Burmese amber has yielded highly diverse and abundant mantispid fossils along with other insect lineages bearing raptorial forelegs, such as the Cretaceous-exclusive Dipteromantispidae and counterparts to the relict Rhachiberothidae, potentially adopting a similar predation strategy (Table S11). Functional trade-offs shaped behavior and prey category discrepancies among sympatric species within the same period, resulting in occupation of differentiated ecological niches [57]. The unique foreleg modification associated with functional and behavioral specialization of Doratomantispinae might have developed for niche differentiation that was stimulated by the intense competition of the Late Cretaceous insect community.

Species distribution modeling and statistical tests

All valid extant and extinct Mantispidae species and their collecting localities were used in the different sets of species distribution modeling (SDM). Distribution of extant species were collected from Lacewing Digital Library [14] (LDL, https://lacewing.tamu.edu). Ages and paleocoordinates of extinct species were collected from LDL and Fossilworks

(http://www.fossilworks.org). Localities were selected for SDM using buffer zone to reduce spatial autocorrelation [58]. Species distribution used in SDM is as in Fig. S27F. Twenty environmental variables were drawn from WorldClim [59] (https://worldclim.org). Ten variables were selected and used in extant SDM after Pearson correlation coefficient analysis (Table S9; Supplementary Data 18). Habitat MAT of extinct species adopted the paleotemperature at each species paleocoordinate and the nearest time in a 5-million-year bin dataset drawn from refs [60,61]. Maps of paleogeography and paleotemperature were derived from refs [62,63] and projected in ArcGIS. Models were evaluated utilizing function 'ENMevaluate' of the R package *ENMeval* v2.0.3 [64]. The optimum model setting was applied in subsequent analyses, using feature classes of LQHP and regularization multiplier as 2.5. SDMs were performed in Maxent v.3.4.4 [65,66] for: i. extant mantispids using ten environmental variables, and ii. extinct and extant mantispids using one variable (mean annual temperature of extinct and extant species distribution of the corresponding time). Statistical analysis of habitat MAT data between extinct and extant species was performed using two-tailed Mann–Whitney *U*-test for non-normal distribution. Lineage number of Mantispidae and Mantodea were calculated from LTT. Correlation between global MAT and lineage number at a 1-million-year time bin were tested using non-parametric Spearman correlation coefficient ρ for non-normal distribution.

Supplementary results and discussions

Plotting extant species distribution on global MAT map illustrates the dense distribution in their favorable temperature zone. We traced their distribution temperature back into geological time by combining extinct species distributions on paleogeography and paleoclimate of corresponding time intervals [60–63]. The results are close for habitat MAT in deep time to the Recent. Two-tailed Mann–Whitney U-test finds no significant difference on habitat MAT data of extinct and extant species (Fig. 1H, Supplementary Data 15).

Supplementary Figures

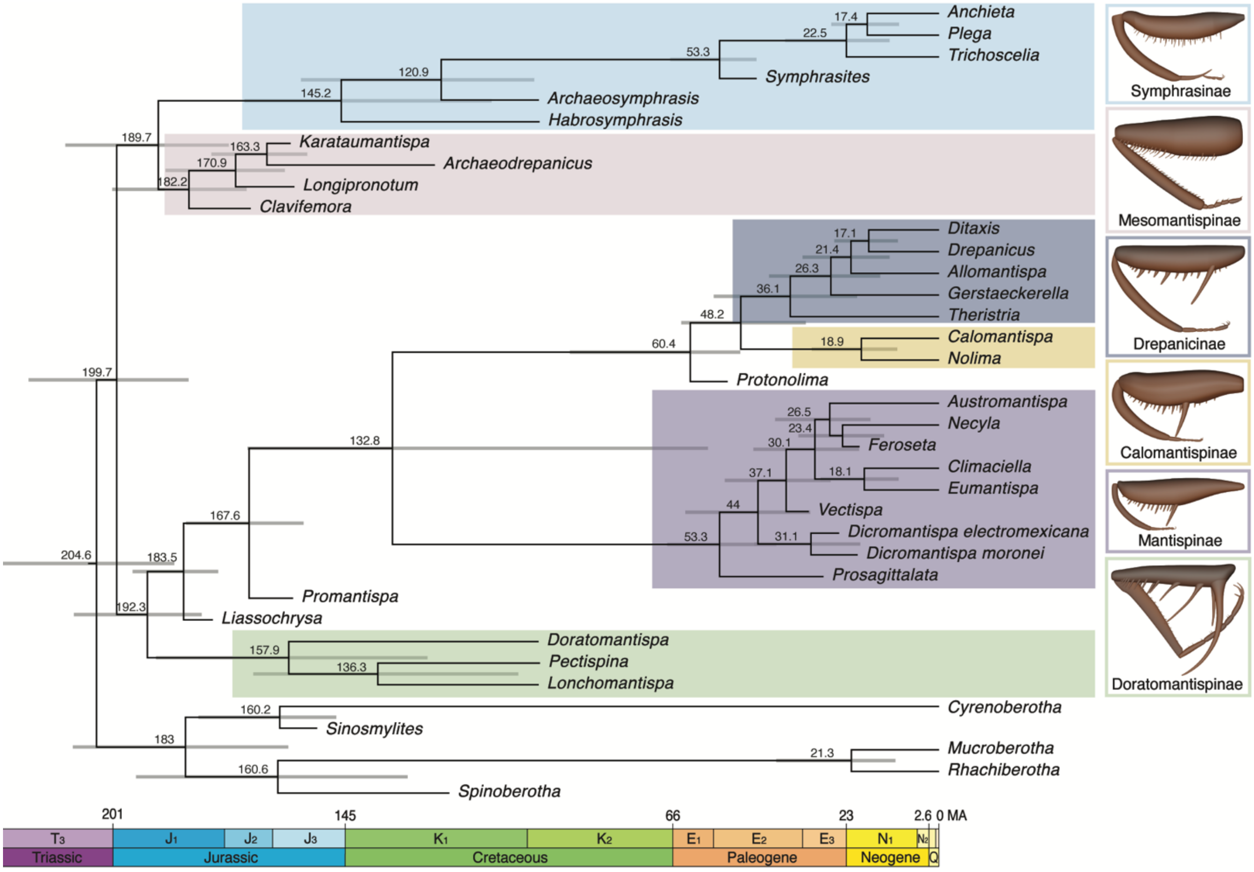

Fig. S1. Raptorial foreleg morphotypes distribution on time-calibrated phylogeny of Mantispidae. Maximum compatible tree from Bayesian phylogenetic tip-dating analysis. The node dates are the median estimates of divergence times. The node bars are age intervals of 95% highest posterior density. Fig. 1B is simplified from this phylogeny.

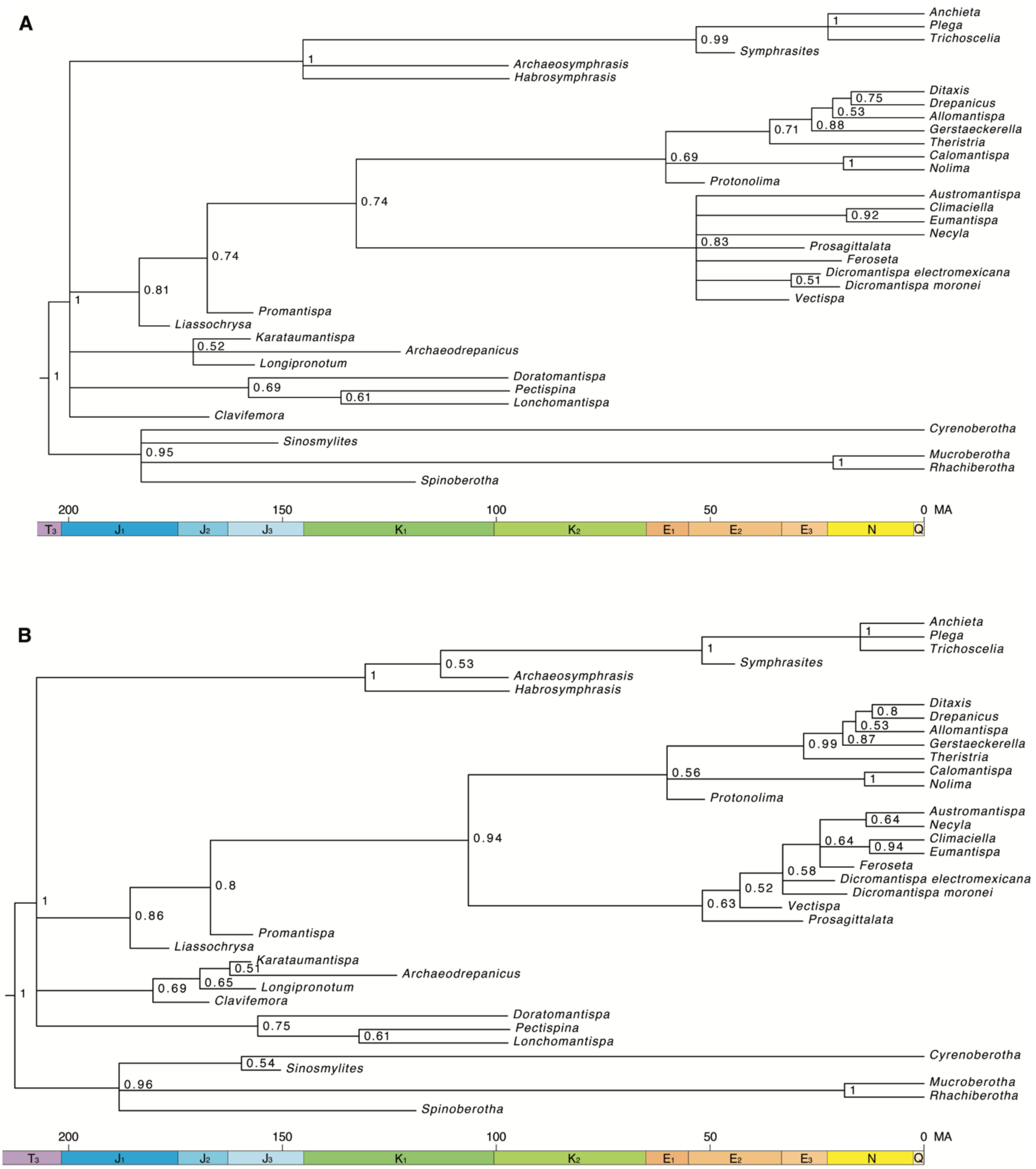

Fig. S2. Posterior consensus tree as a 50% majority-rule using (A) a single morphological clock partition, (B) three-partitioned morphological clocks.

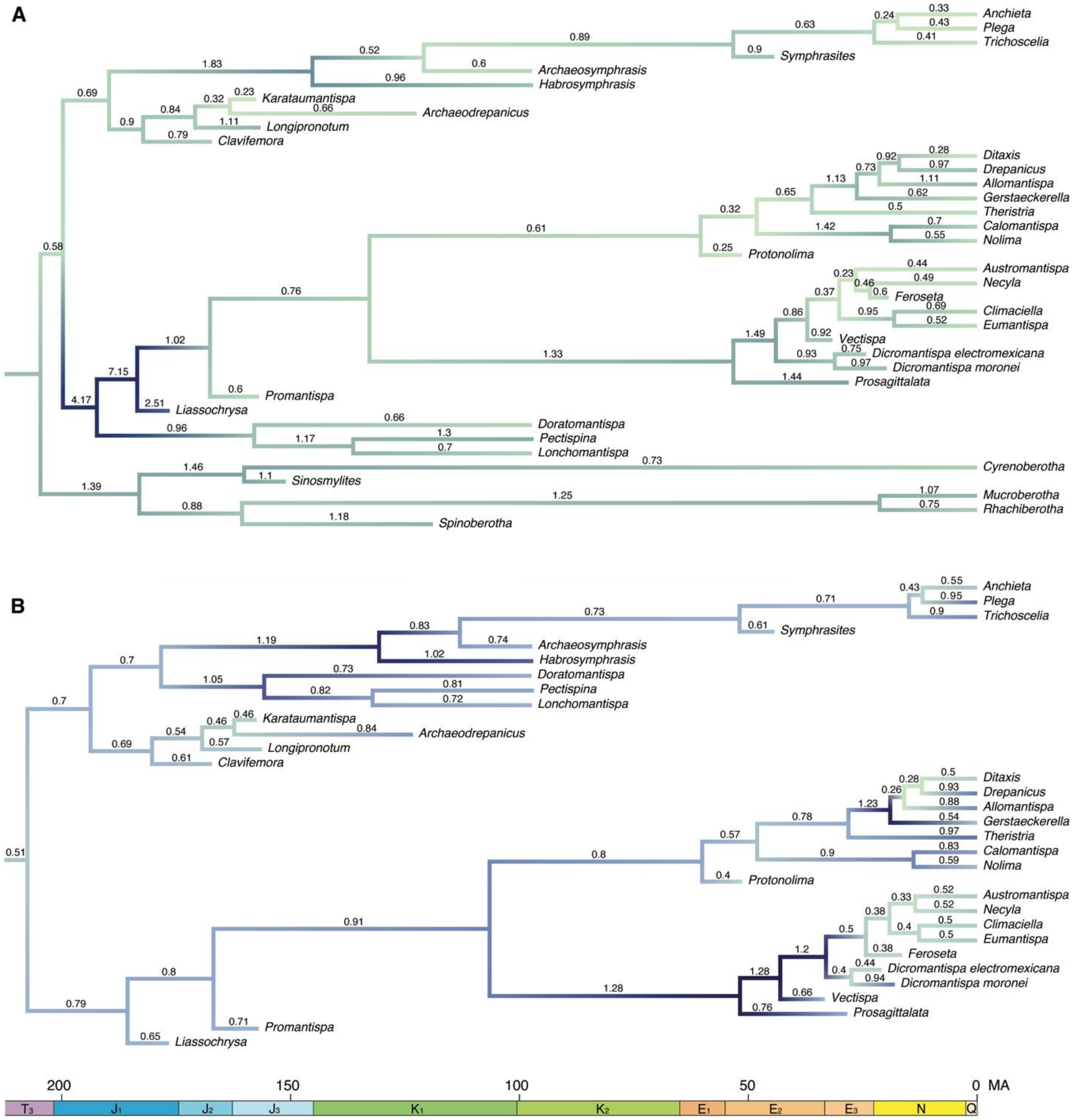

Fig. S3. (A) Median rates of overall morphological evolution obtained from tip dating analysis using a single morphological clock partition; (B) Median rates of foreleg morphological evolution of ingroups obtained from tip dating analysis using three-partitioned morphological clocks.

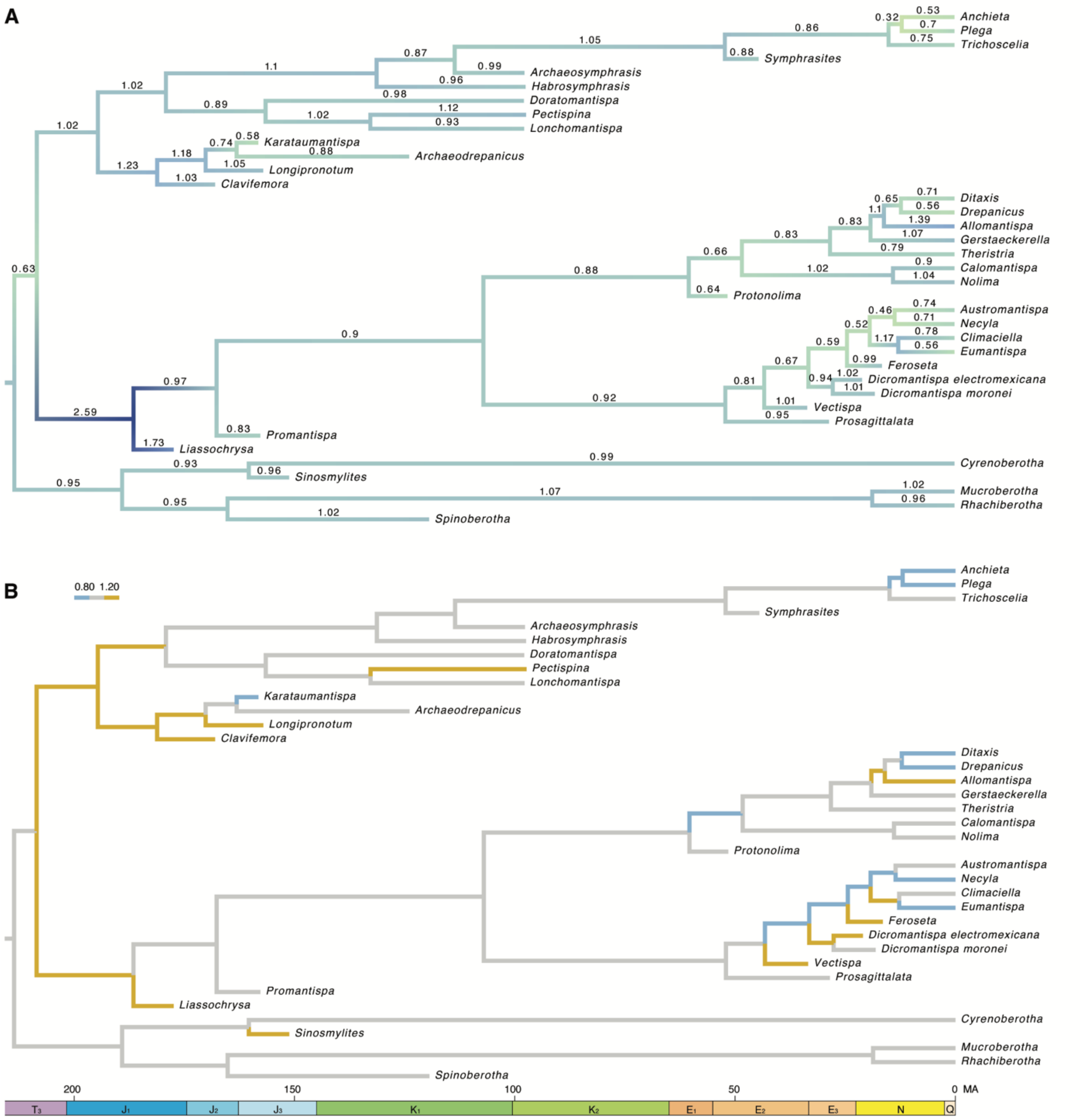

Fig. S4. (A) Median rates of forewing morphological evolution and (B) forewing relative rates obtained from tip dating analysis using three-partitioned morphological clocks.

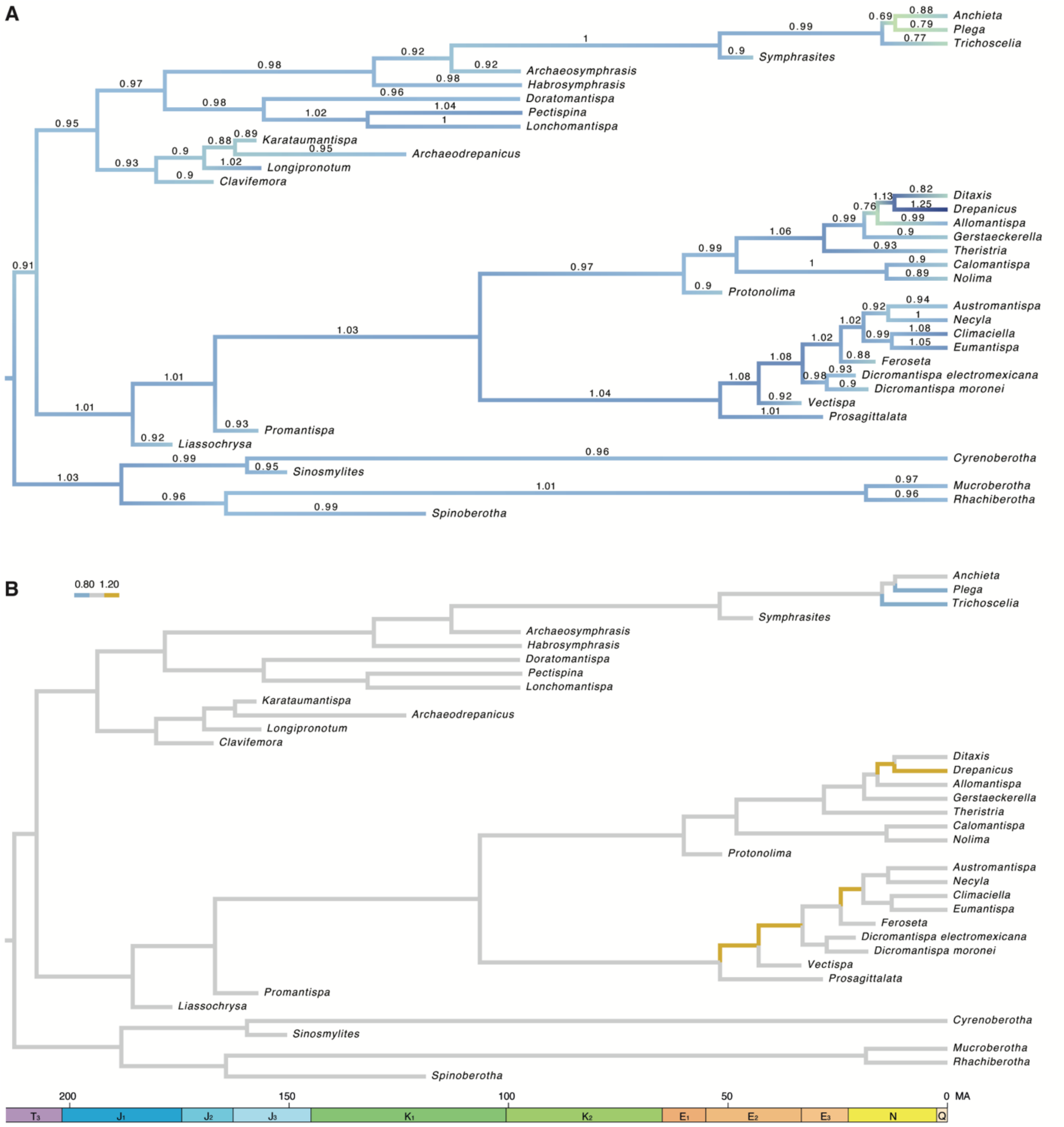

Fig. S5. (A) Median rates of morphological evolution of other body parts and (B) relative rates obtained from tip dating analysis using three-partitioned morphological clocks.

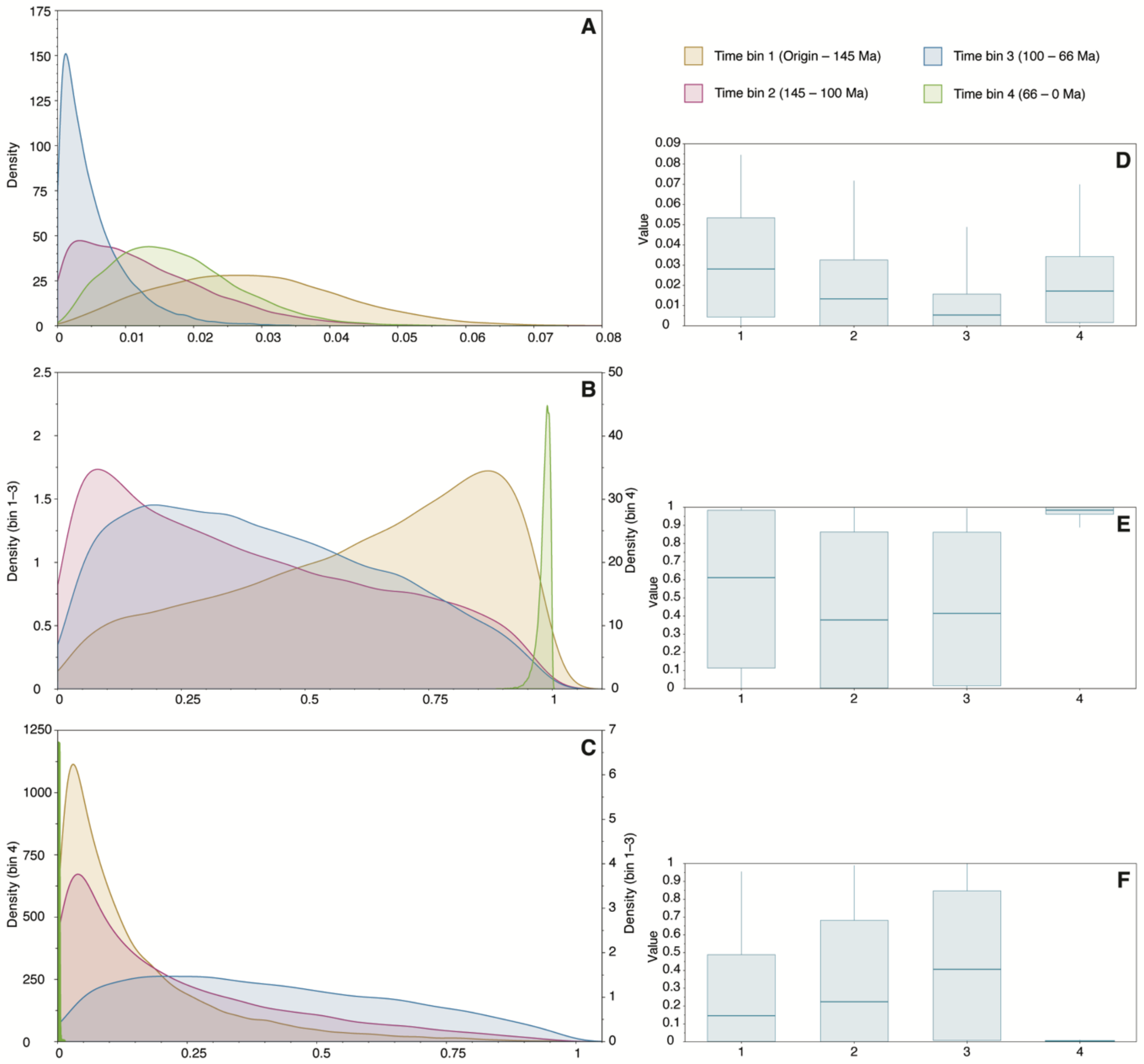

Fig. S6. Macroevolutionary parameter estimates across time bins. Kernel density estimation of (**A**) net diversification, (**B**) relative extinction, (**C**) relative fossilization; mean and quartile values of (**D**) net diversification, (**E**) relative extinction, (**F**) relative fossilization in each time bin.

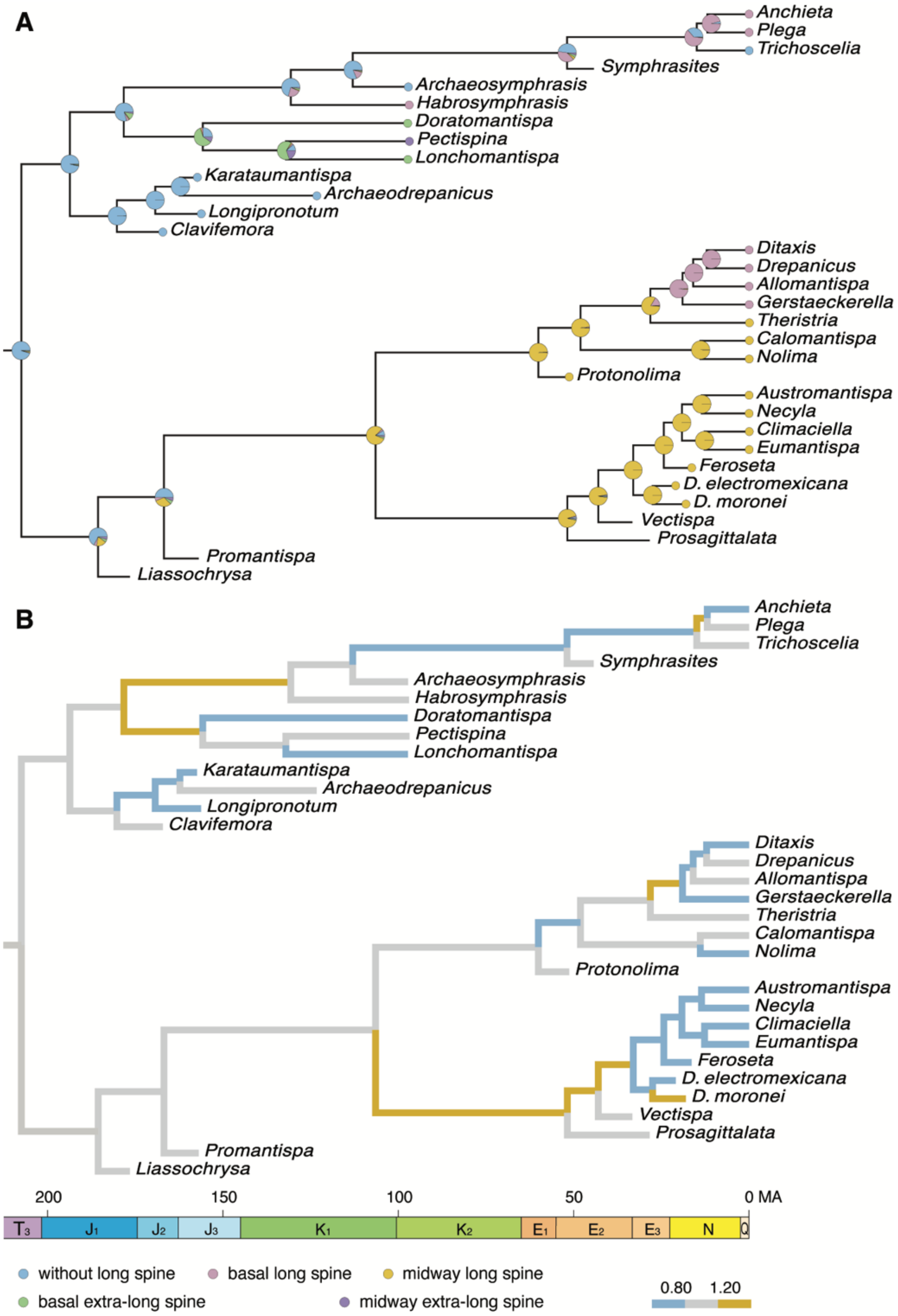

Fig. S7. Ancestral state reconstruction and relative evolutionary rates of foreleg in Mantispidae. (**A**) Ancestral state reconstruction for forefemoral major spine evolution and (**B**) foreleg relative rates of ingroups mapped on tip-dating phylogenetic tree using partitioned morphological clocks.

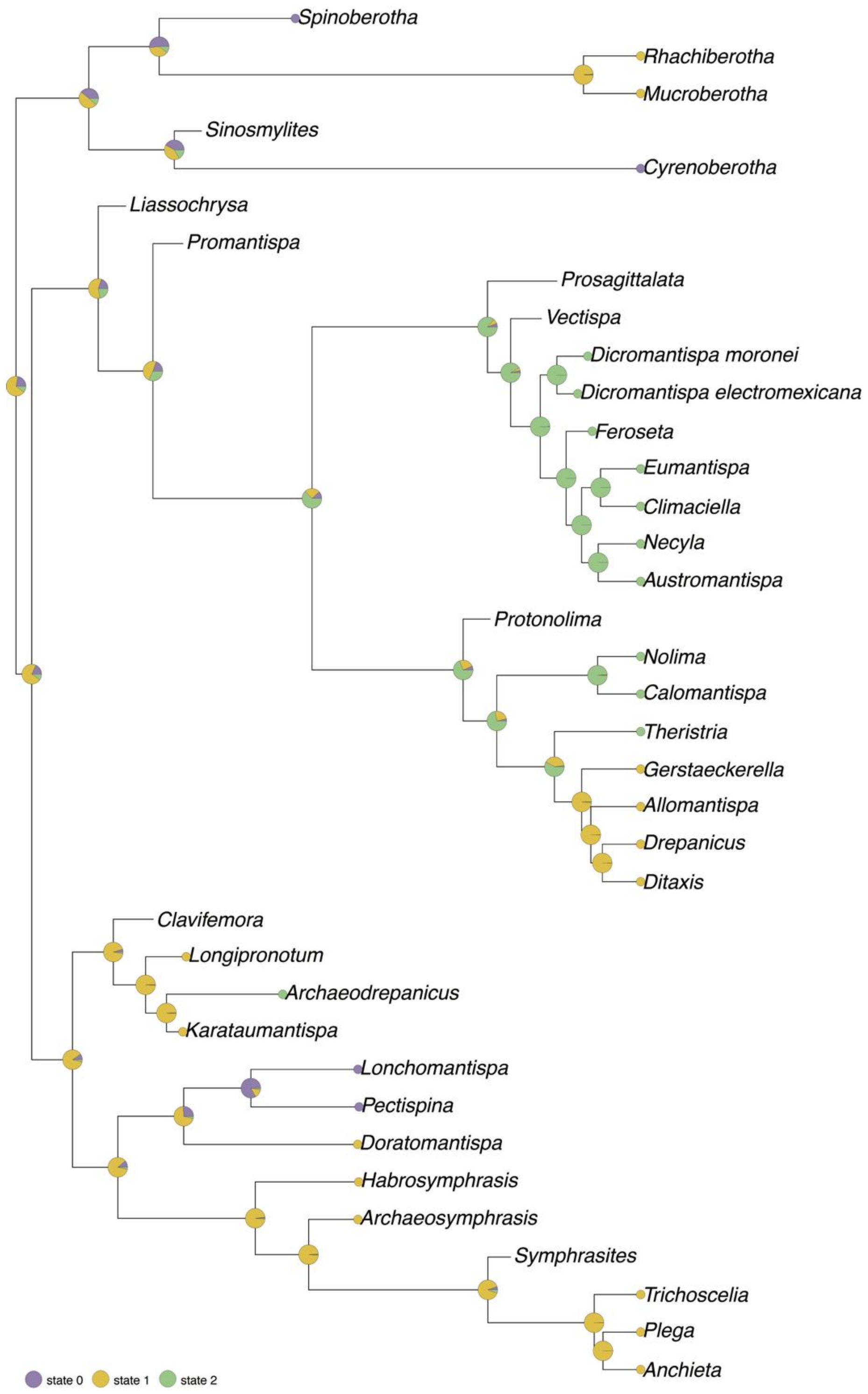

Fig. S8. Ancestral state reconstruction of forefemur length compared to foretibia and foretarsi together. State 0, significantly shorter; 1, subequal; 2, longer.

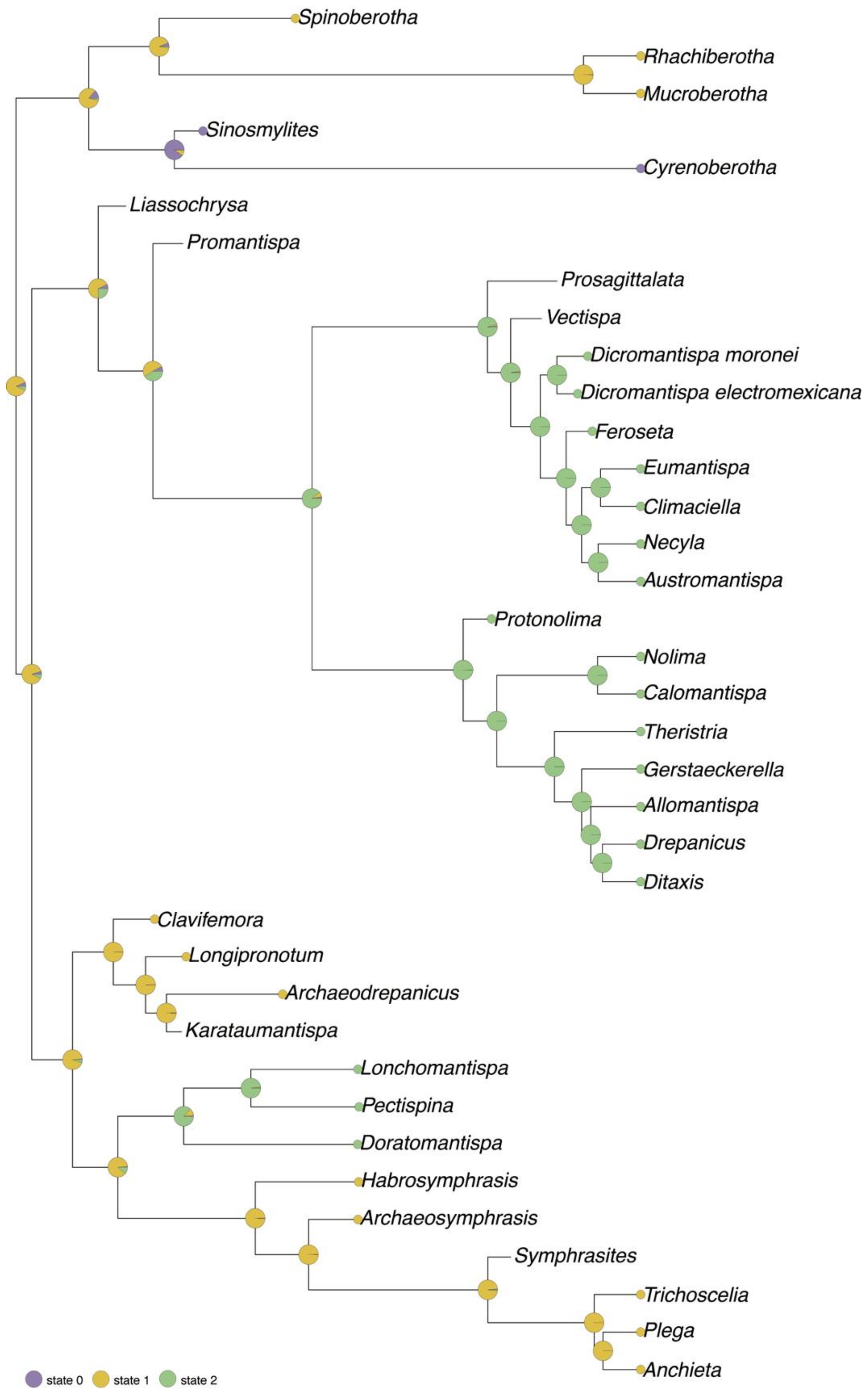

Fig. S9. Ancestral state reconstruction of forefemoral appendage. State 0, thin setae; 1, spine-like setae; 2, cuticular spine.

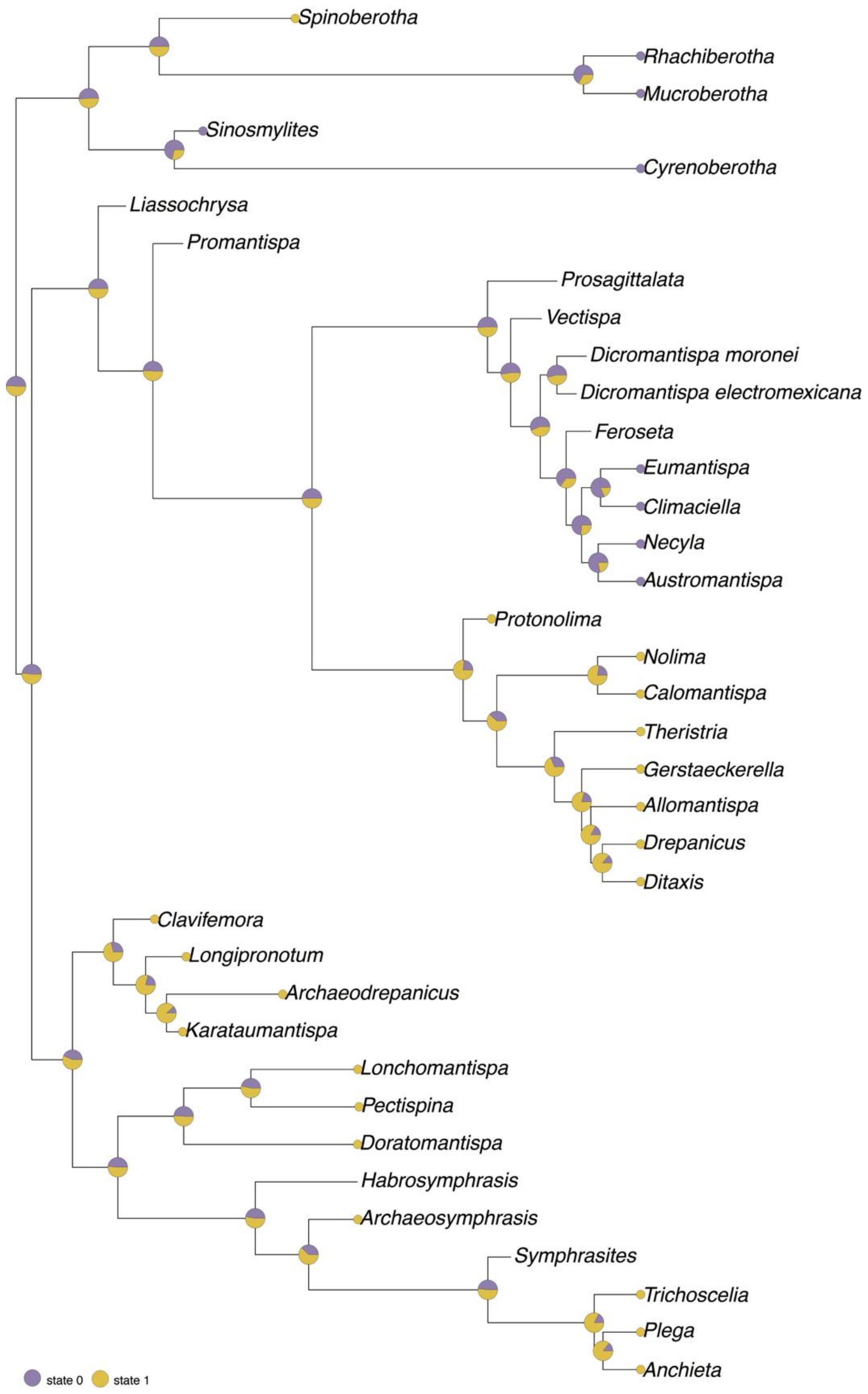

Fig. S10. Ancestral state reconstruction of foretibial ventral appendage. State 0, thin setae; 1, thick prostrate setae.

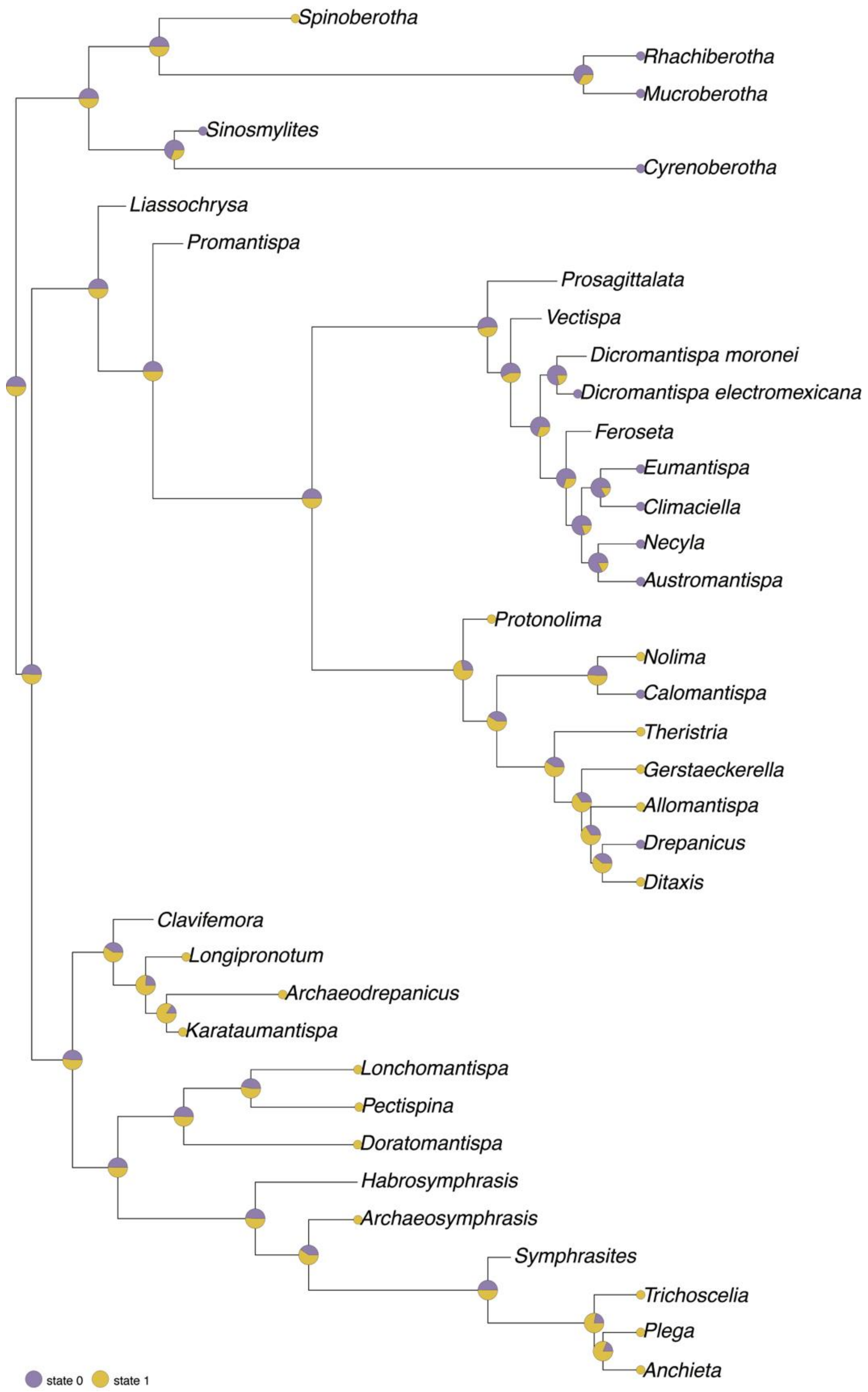

Fig. S11. Ancestral state reconstruction of foretarsal ventral appendage. State 0, thin setae; 1, thick prostrate setae.

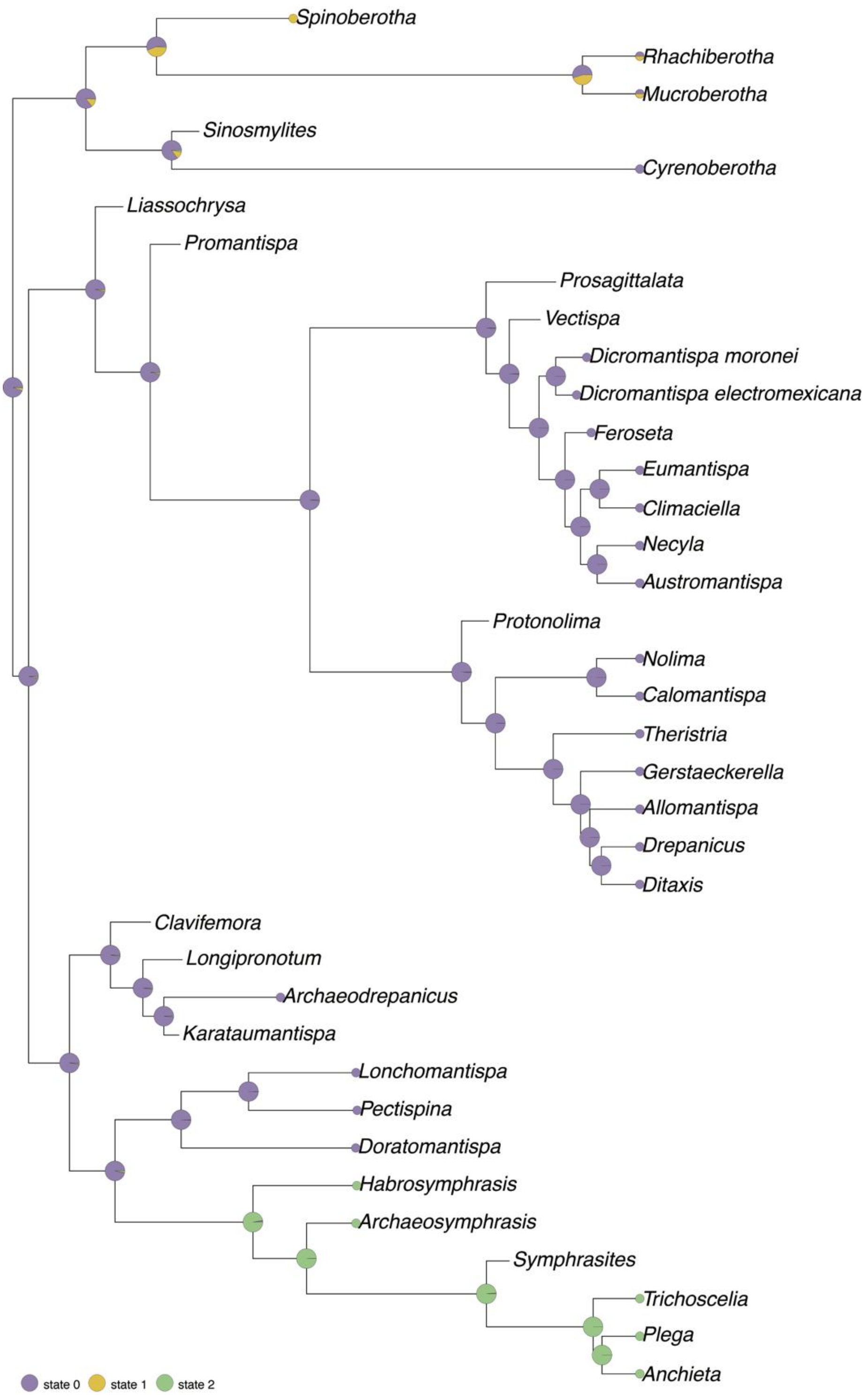

Fig. S12. Ancestral state reconstruction of foretarsus first element apex. State 0, short thin setae; 1, long spine-like setae; 2, long cuticular setae.

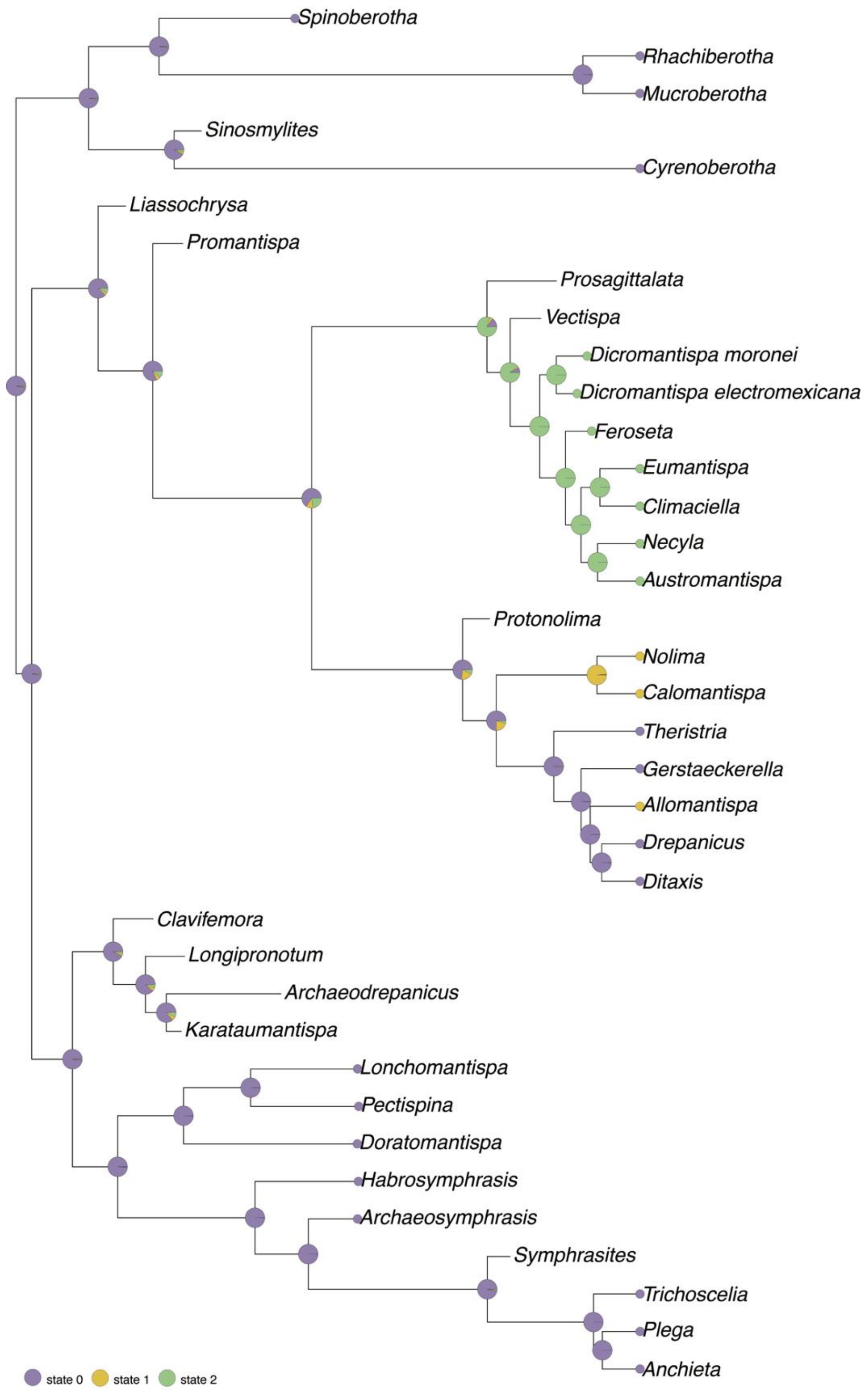

Fig. S13. Ancestral state reconstruction of foretarsal claw. State 0, two simple; 1, two bifid; 2, one simple.

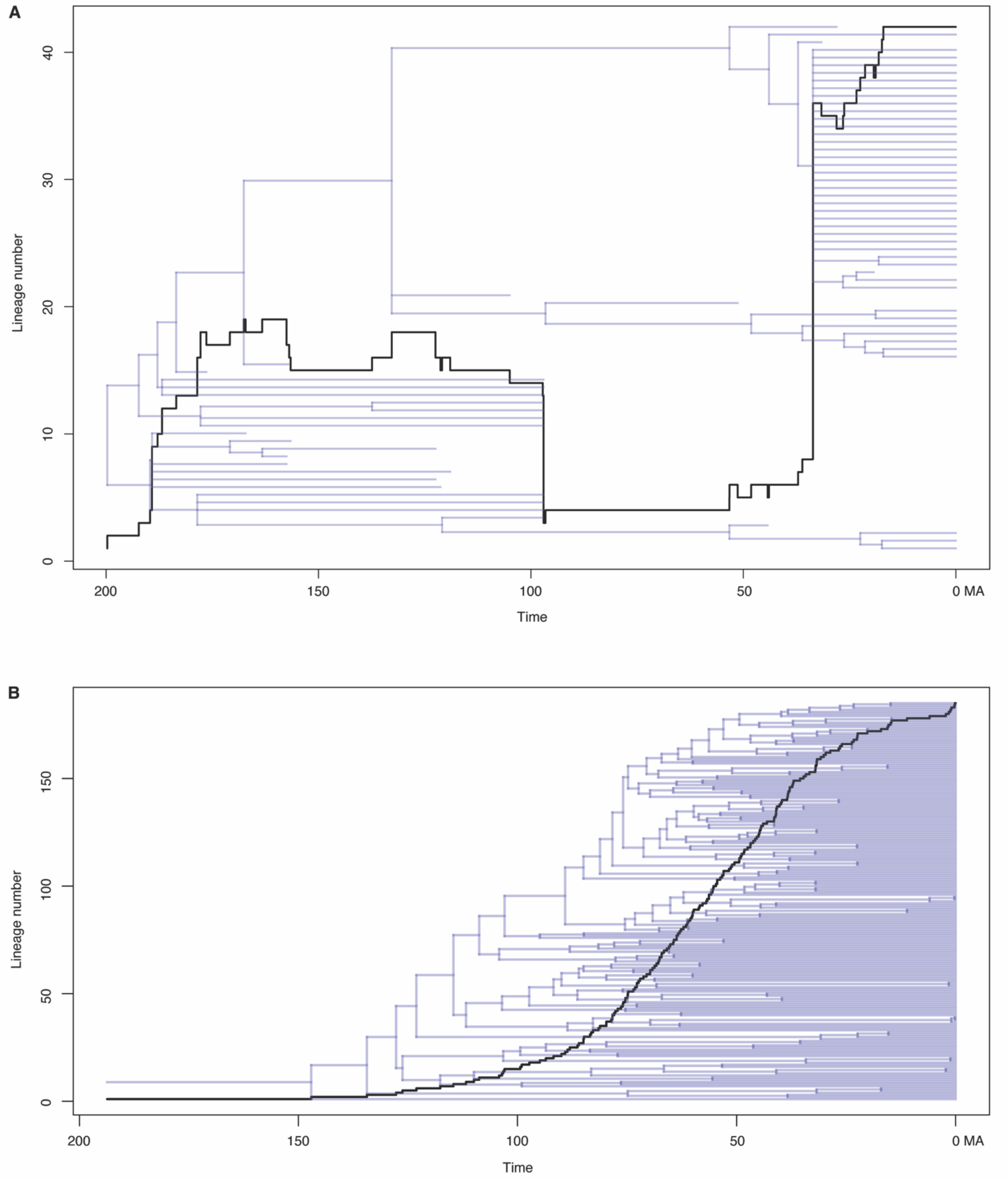

Fig. S14. (A) LTT plot of Mantispidae in linear scale derived from the time tree in the background. (B) LTT of Mantodea in linear scale derived from the time tree in the background.

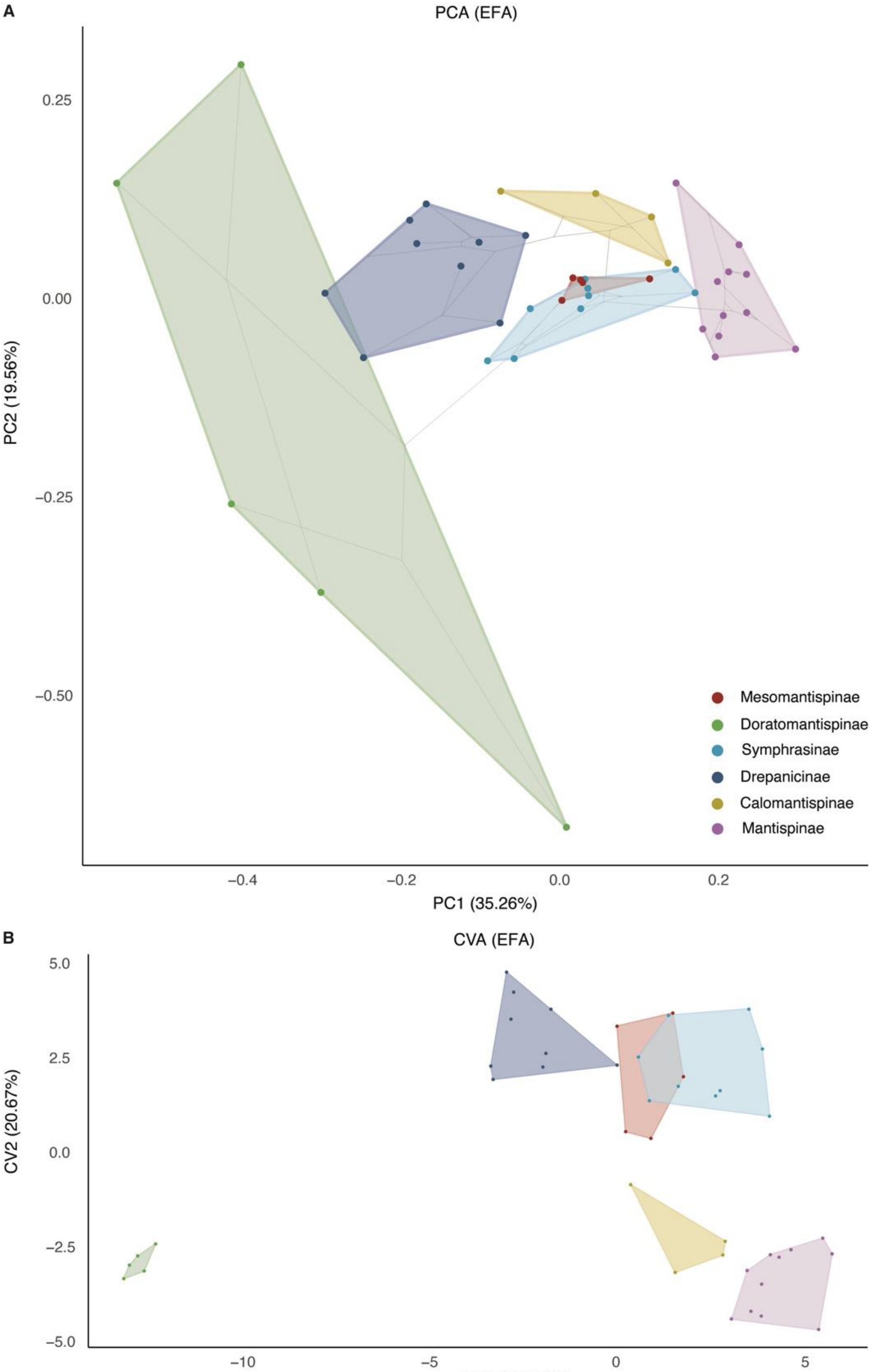

Fig. S15. (A) Phylomorphospace of forefemora outlines by EFA. Fig. 1A is modified from this result by plotting chronologically. (B) CVA from first 20 PCs.

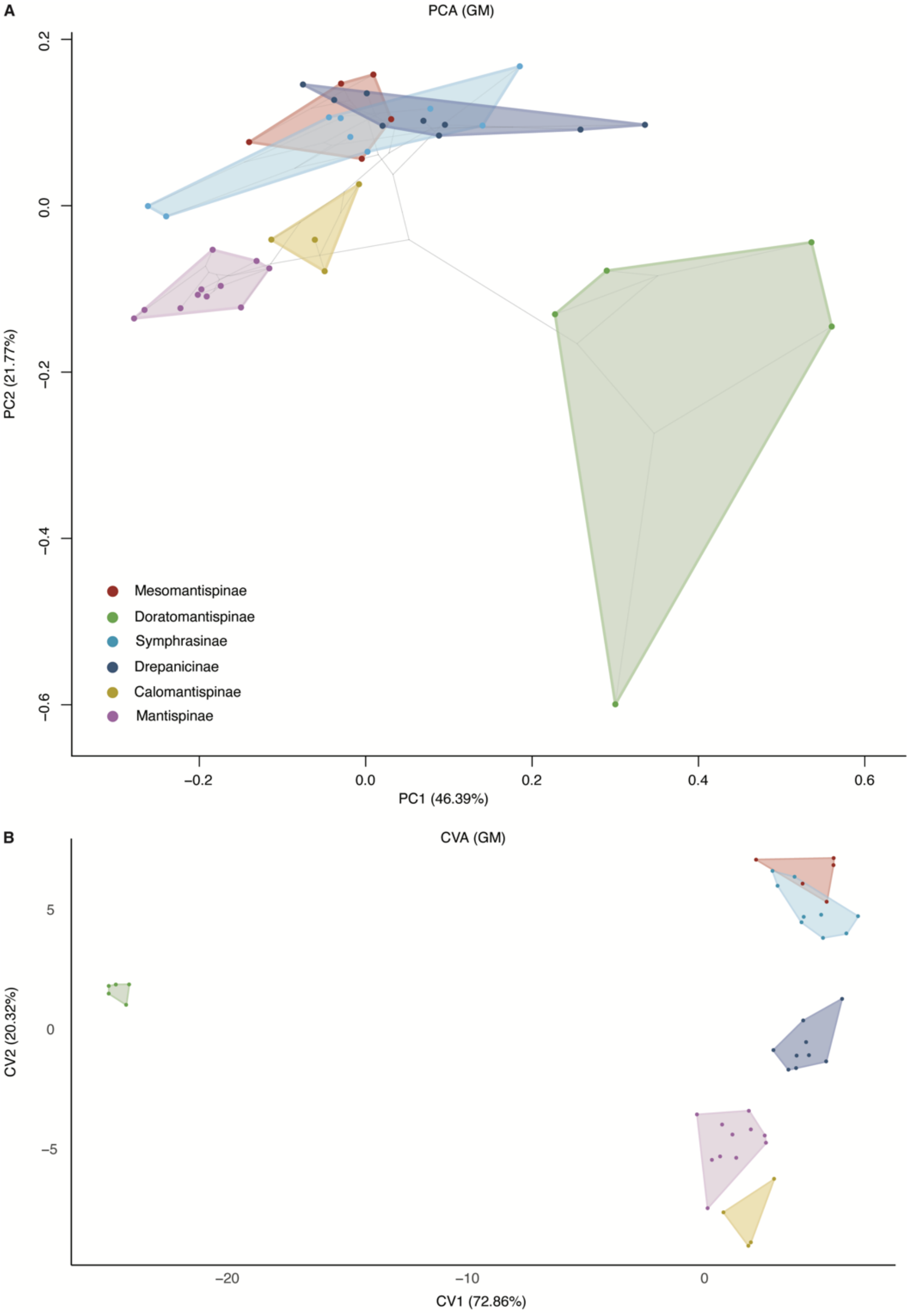

Fig. S16. (A) Phylomorphospace of forefemora outlines by GM and (B) CVA from first 20 PCs.

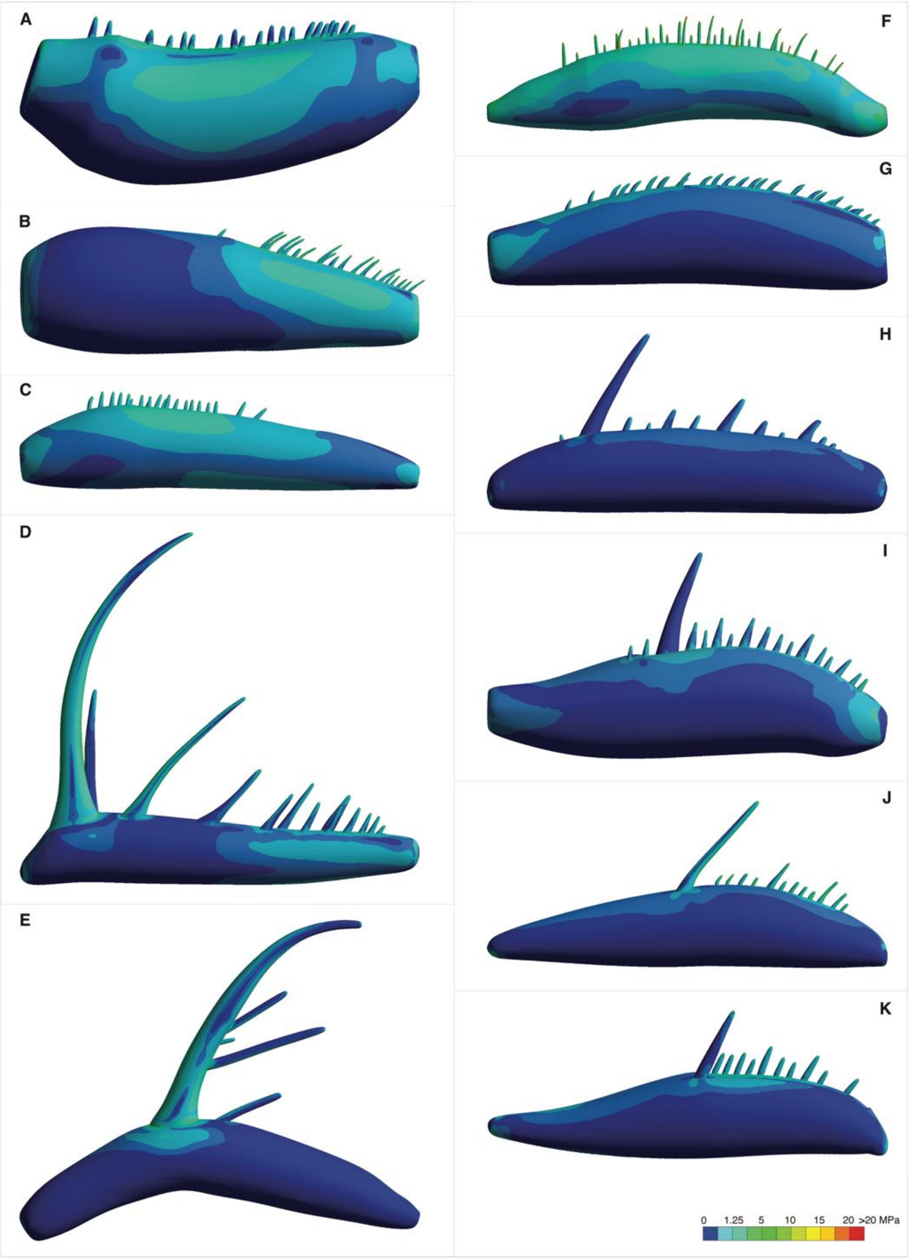

Fig. S17. FEA along single femoral spine with loading of 0.001 N. (**A**) *Clavifemora rotundata*; (**B**) *Longipronotum benmaddoxi*; (**C**) *Archaeodrepanicus nuddsi*; (**D**) *Lonchomantispa longa*; (**E**) *Pectispina libera*; (**F**) *Archaeosymphrasis pennyi*; (**G**) *Trichoscelia varia*; (**H**) *Drepanicus gayi*; (**I**) *Nolima victor*; (**J**) *Dicromantispa electromexicana*; (**K**) *Necyla minor*.

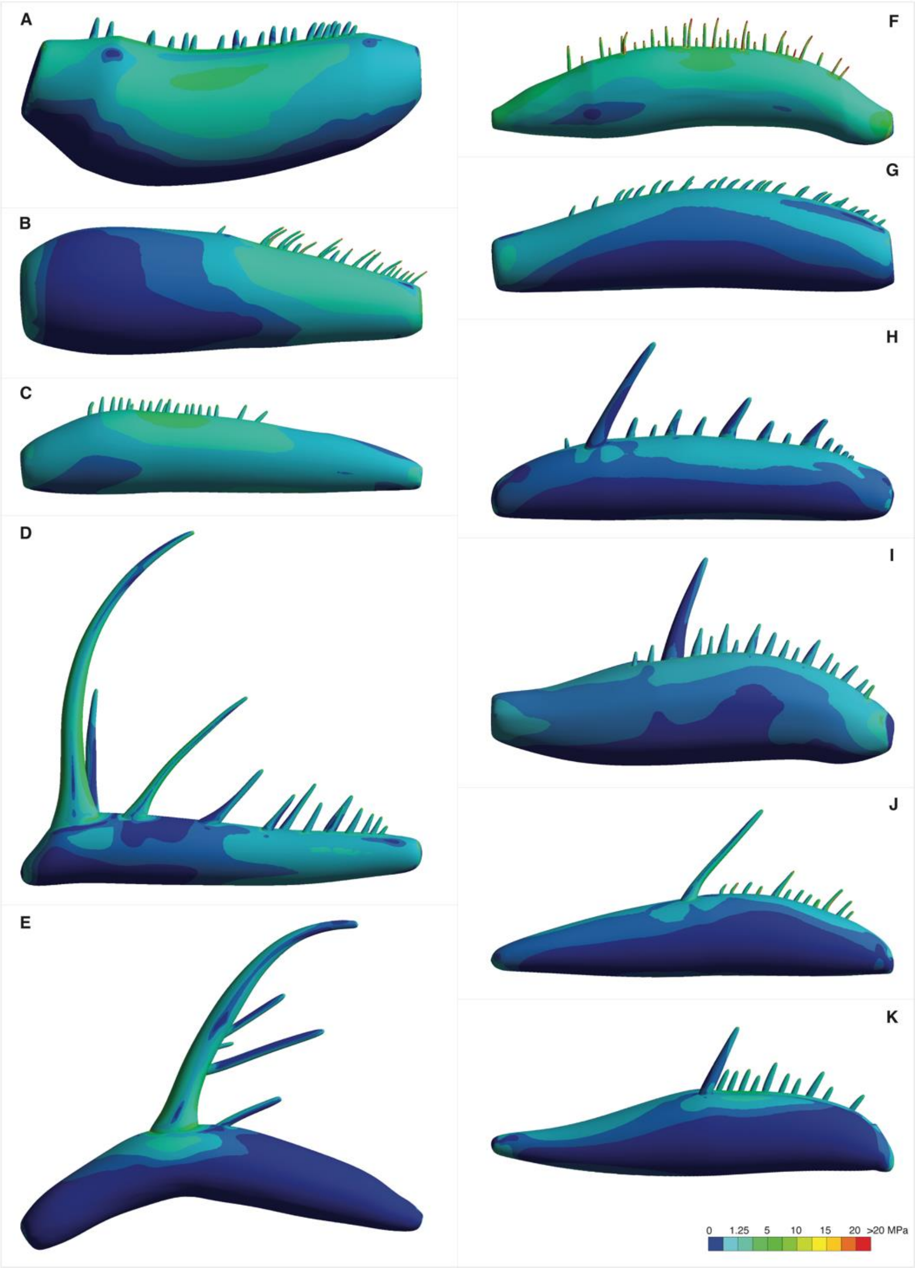

Fig. S18. FEA along single femoral spine with loading of 0.0015 N. (**A**) *Clavifemora rotundata*; (**B**) *Longipronotum benmaddoxi*; (**C**) *Archaeodrepanicus nuddsi*; (**D**) *Lonchomantispa longa*; (**E**) *Pectispina libera*; (**F**) *Archaeosymphrasis pennyi*; (**G**) *Trichoscelia varia*; (**H**) *Drepanicus gayi*; (**I**) *Nolima victor*; (**J**) *Dicromantispa electromexicana*; (**K**) *Necyla minor*.

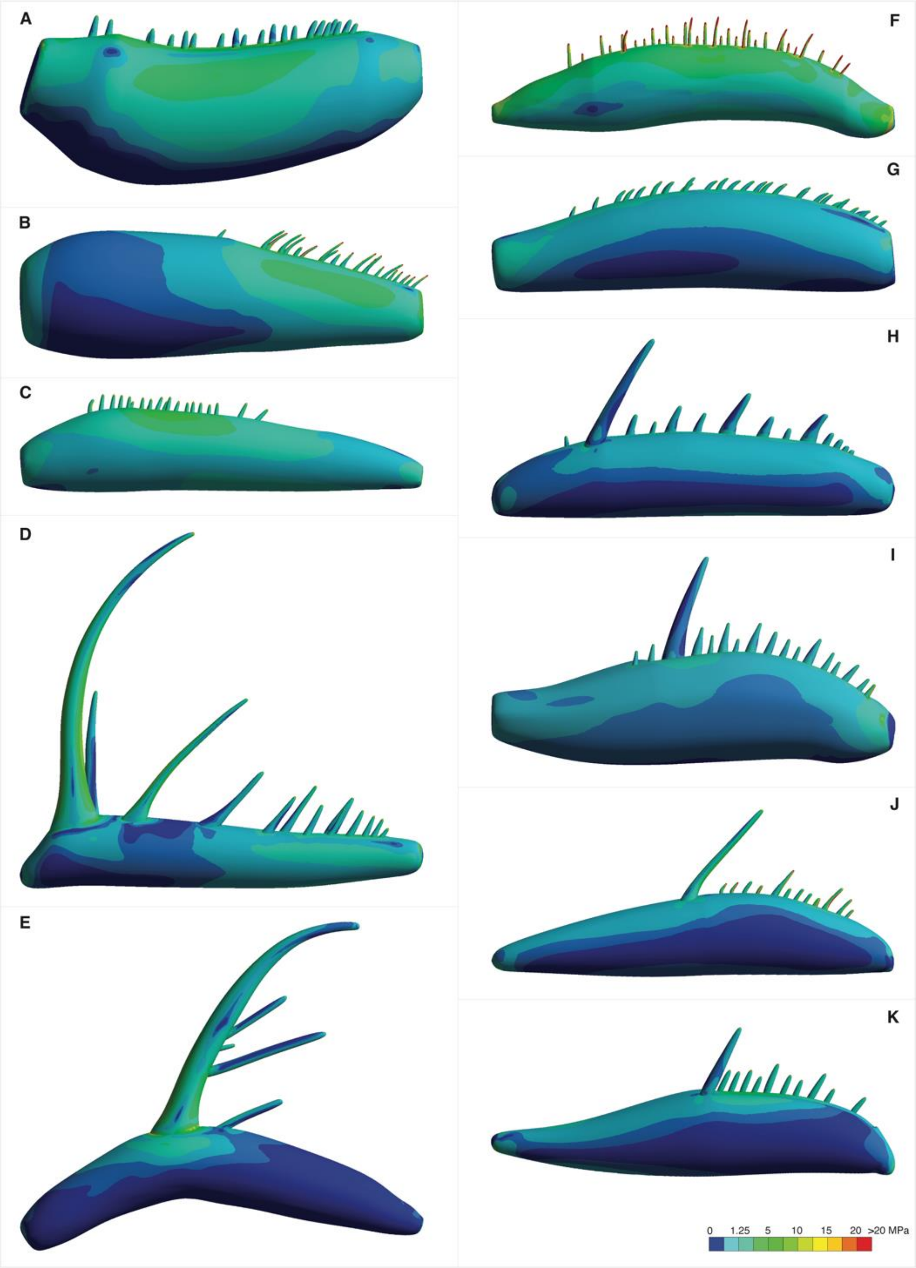

Fig. S19. FEA along single femoral spine with loading of 0.002 N. (**A**) *Clavifemora rotundata*; (**B**) *Longipronotum benmaddoxi*; (**C**) *Archaeodrepanicus nuddsi*; (**D**) *Lonchomantispa longa*; (**E**) *Pectispina libera*; (**F**) *Archaeosymphrasis pennyi*; (**G**) *Trichoscelia varia*; (**H**) *Drepanicus gayi*; (**I**) *Nolima victor*; (**J**) *Dicromantispa electromexicana*; (**K**) *Necyla minor*.

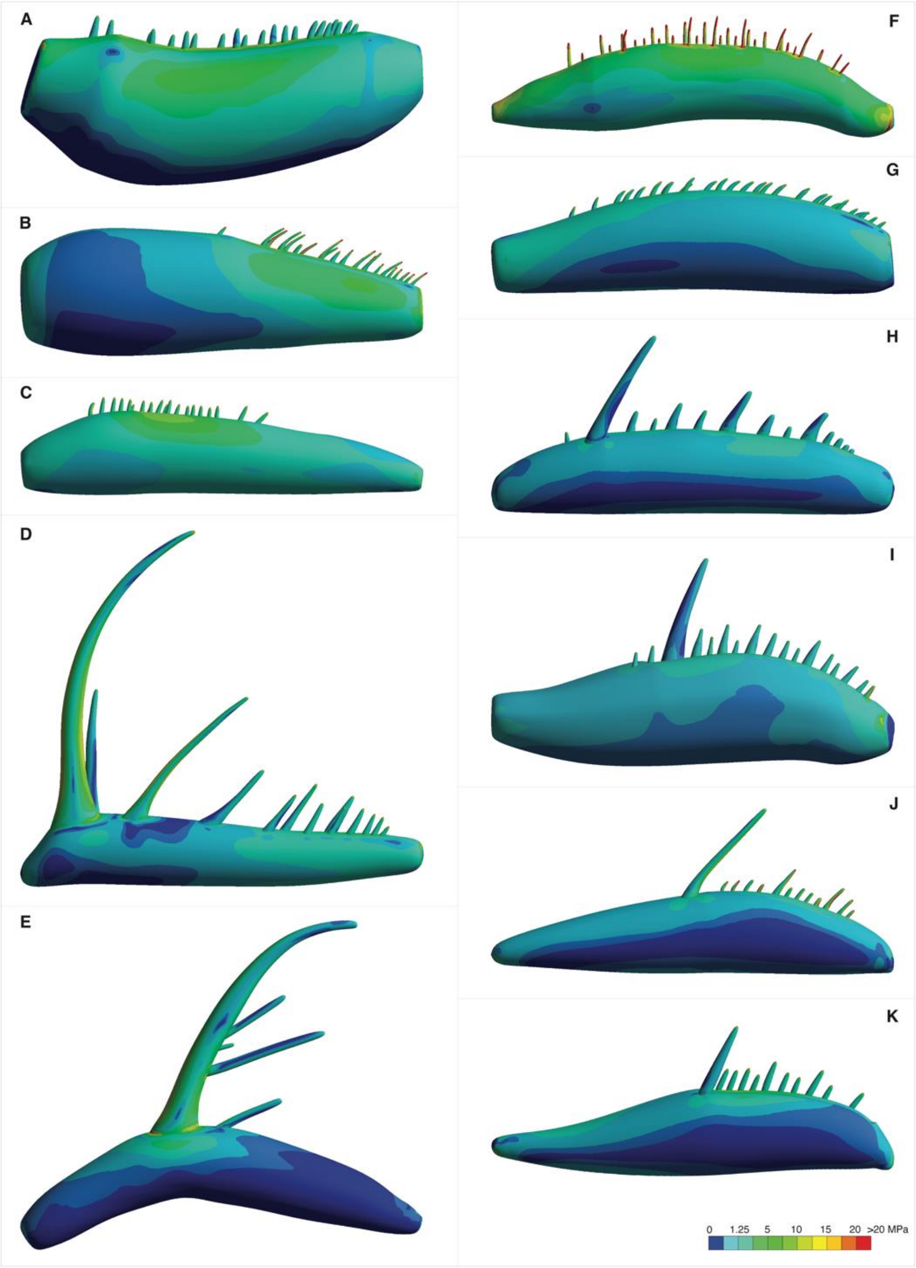

Fig. S20. FEA along single femoral spine with loading of 0.0025 N. (**A**) *Clavifemora rotundata*; (**B**) *Longipronotum benmaddoxi*; (**C**) *Archaeodrepanicus nuddsi*; (**D**) *Lonchomantispa longa*; (**E**) *Pectispina libera*; (**F**) *Archaeosymphrasis pennyi*; (**G**) *Trichoscelia varia*; (**H**) *Drepanicus gayi*; (**I**) *Nolima victor*; (**J**) *Dicromantispa electromexicana*; (**K**) *Necyla minor*.

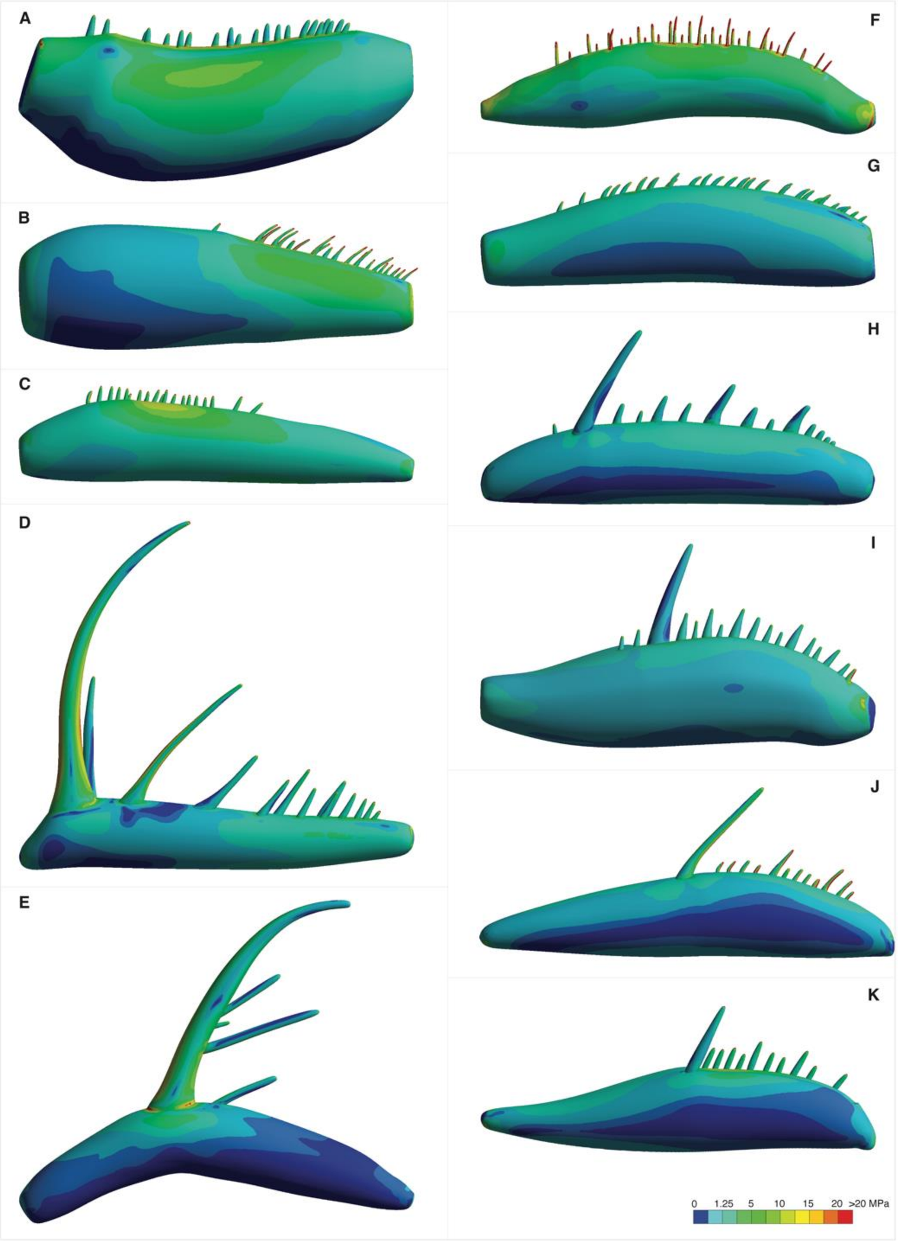

Fig. S21. FEA along single femoral spine with loading of 0.003 N. (**A**) *Clavifemora rotundata*; (**B**) *Longipronotum benmaddoxi*; (**C**) *Archaeodrepanicus nuddsi*; (**D**) *Lonchomantispa longa*; (**E**) *Pectispina libera*; (**F**) *Archaeosymphrasis pennyi*; (**G**) *Trichoscelia varia*; (**H**) *Drepanicus gayi*; (**I**) *Nolima victor*; (**J**) *Dicromantispa electromexicana*; (**K**) *Necyla minor*.

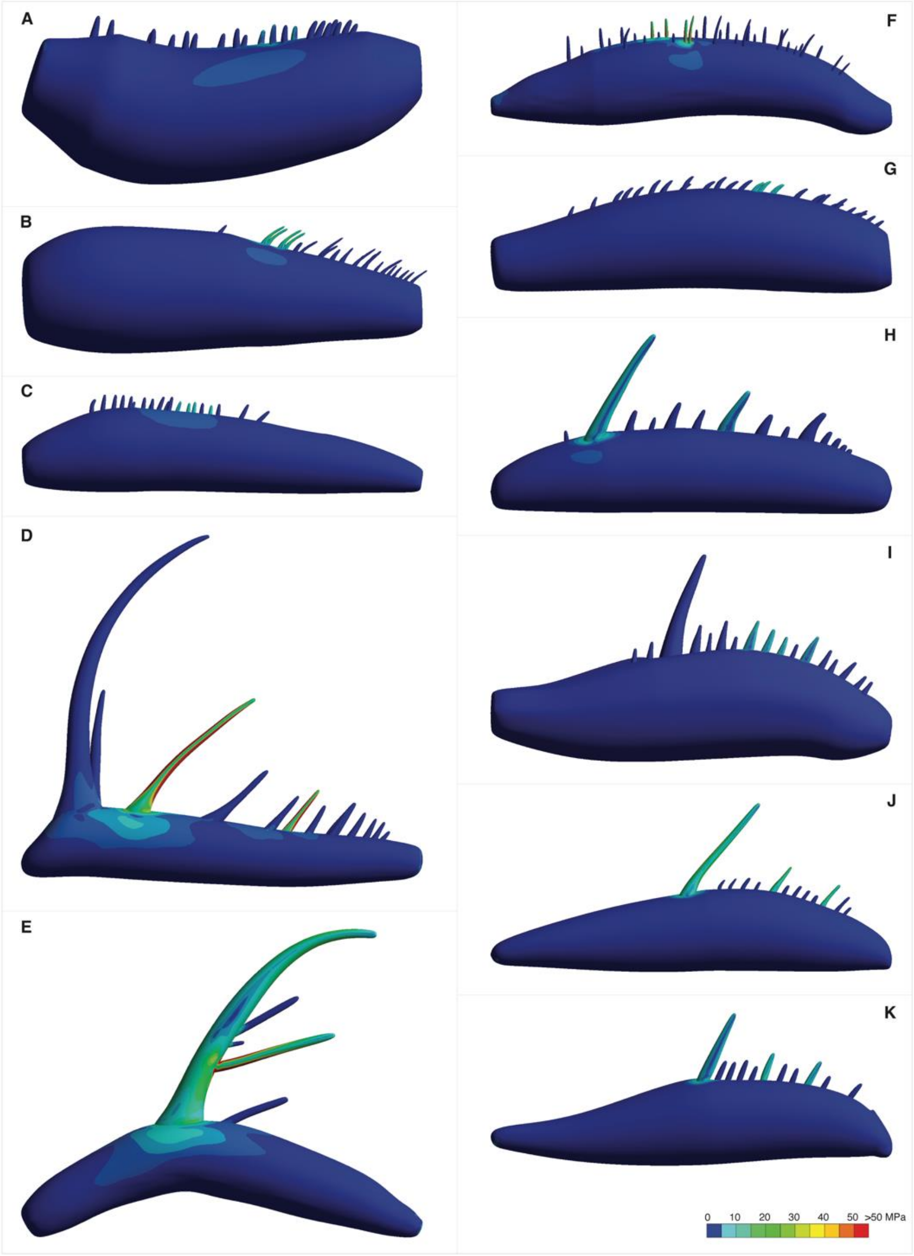

Fig. S22. FEA in simulation of clamping rigid surfaced prey with a total load of 0.006 N on foretibia. (**A**) *Clavifemora rotundata*; (**B**) *Longipronotum benmaddoxi*; (**C**) *Archaeodrepanicus nuddsi*; (**D**) *Lonchomantispa longa*; (**E**) *Pectispina libera*; (**F**) *Archaeosymphrasis pennyi*; (**G**) *Trichoscelia varia*; (**H**) *Drepanicus gayi*; (**I**) *Nolima victor*; (**J**) *Dicromantispa electromexicana*; (**K**) *Necyla minor*.

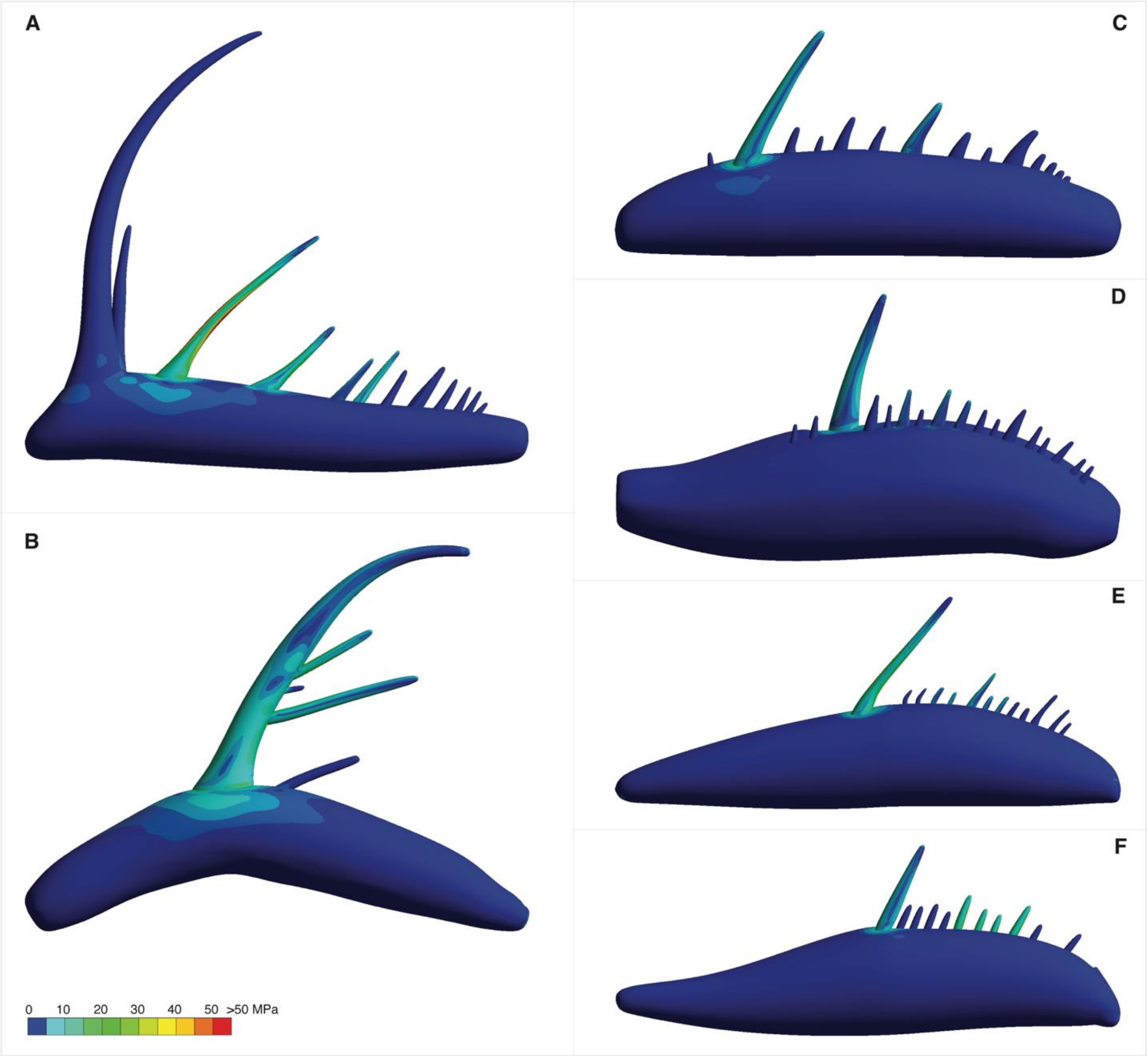

Fig. S23. FEA in simulation of piercing penetrable surfaced prey with a total load of 0.006 N on foretibia. (**A**) *Lonchomantispa longa*; (**B**) *Pectispina libera*; (**C**) *Drepanicus gayi*; (**D**) *Nolima victor*; (**E**) *Dicromantispa electromexicana*; (**F**) *Necyla minor*.

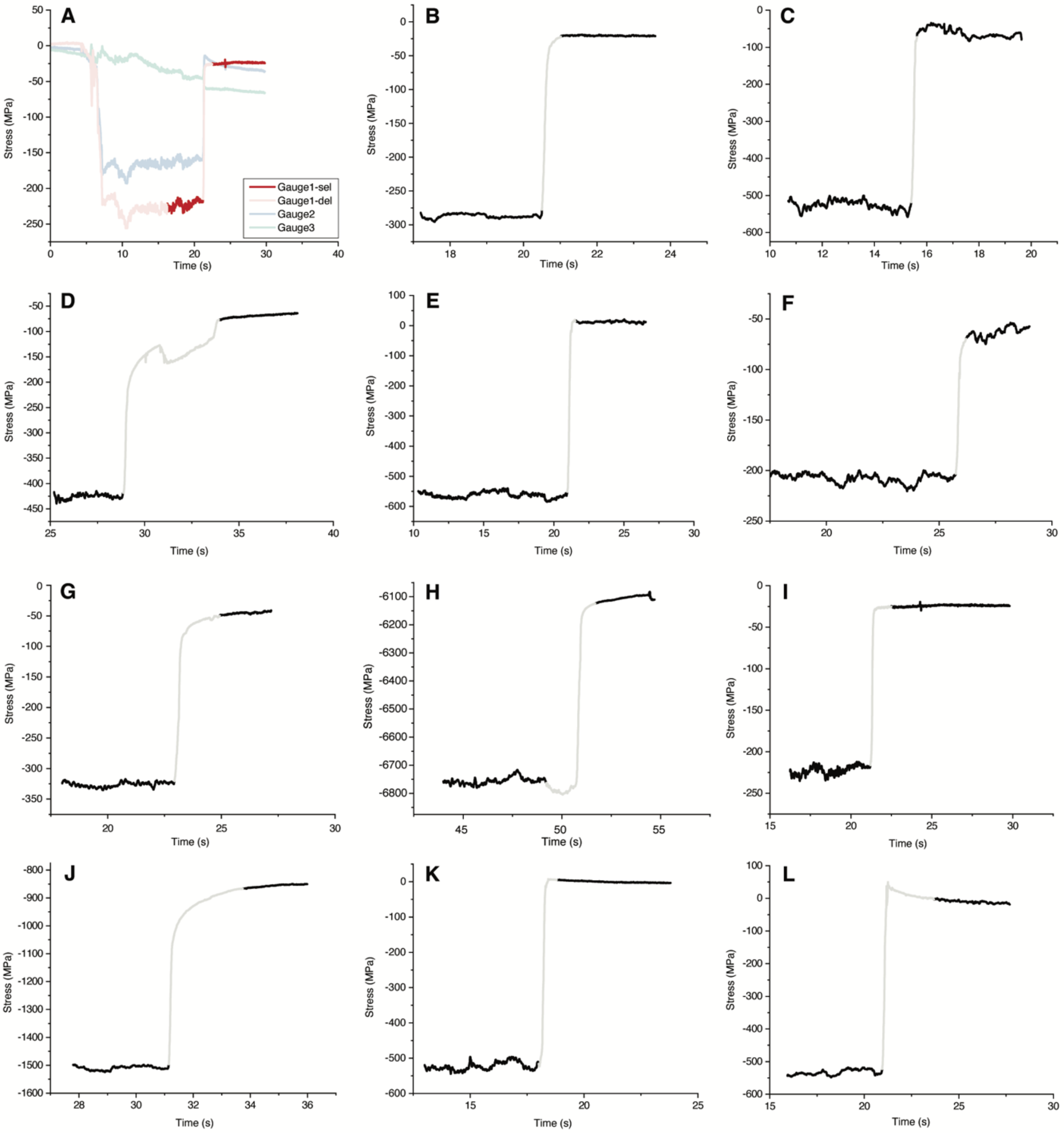

Fig. S24. Stress curves in mechanical experiments. (**A**) stress data selection criterion in mechanical experiments simulating clamping prey; (**B** to **L**) extracted time bins and respective data of (B) *Clavifemora rotundata*, (C) *Longipronotum benmaddoxi*, (D) *Archaeodrepanicus nuddsi*, (E) *Lonchomantispa longa*, (F) *Pectispina libera*, (G) *Archaeosymphrasis pennyi*, (H) *Trichoscelia varia*, (I) *Drepanicus gayi*, (J) *Nolima victor*, (K) *Dicromantispa electromexicana*, (L) *Necyla minor*.

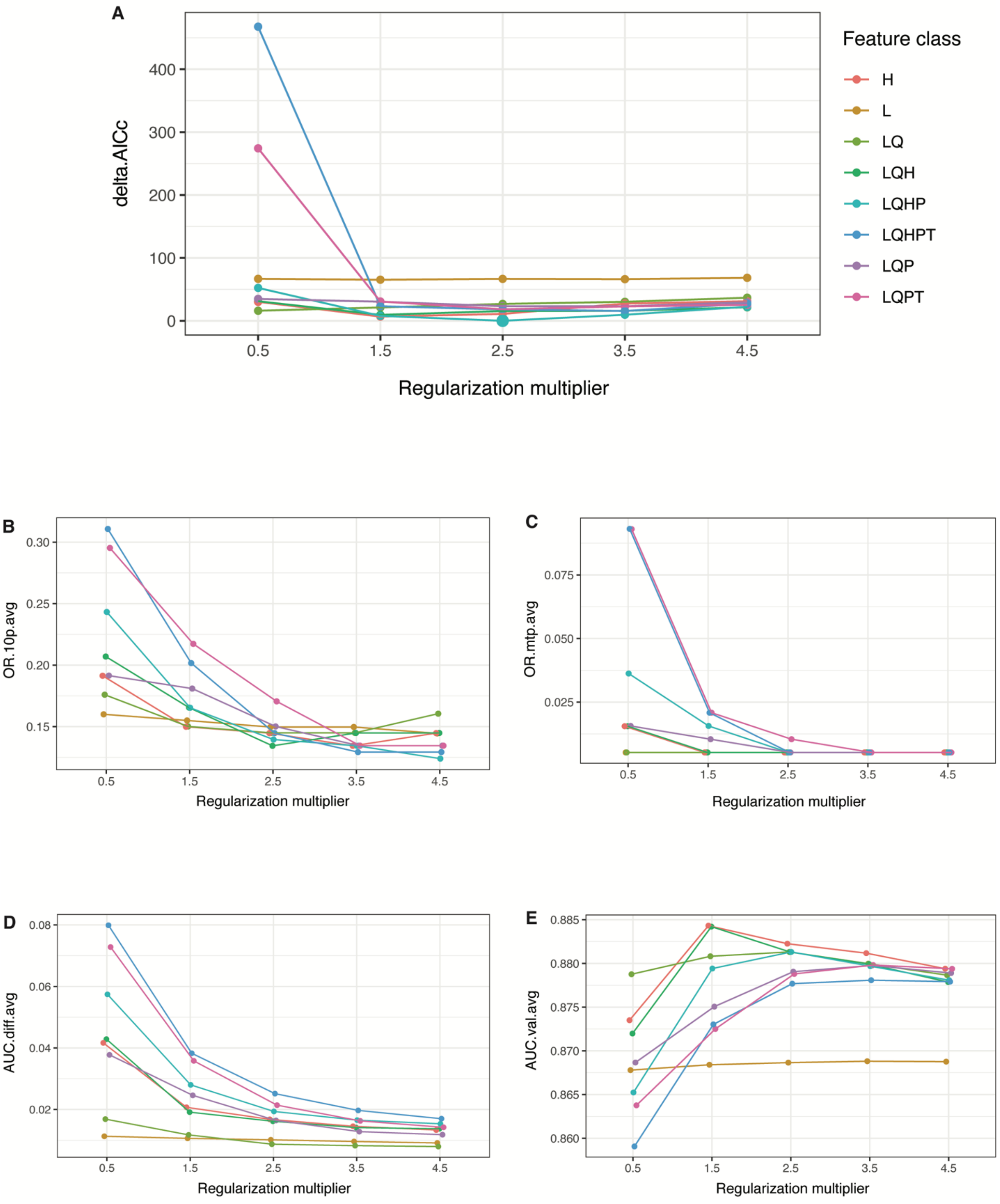

Fig. S25. Evaluation metrics of Maxent models across feature class combinations and regularization multipliers. (**A**) ΔAICc; (**B**) OR_{10P}; (**C**) OR_{MTP}; (**D**) AUC_{DIFF}; (**E**) AUC_{VAL}; H, hinge; L, linear; Q, quadratic; P, product; T, threshold.

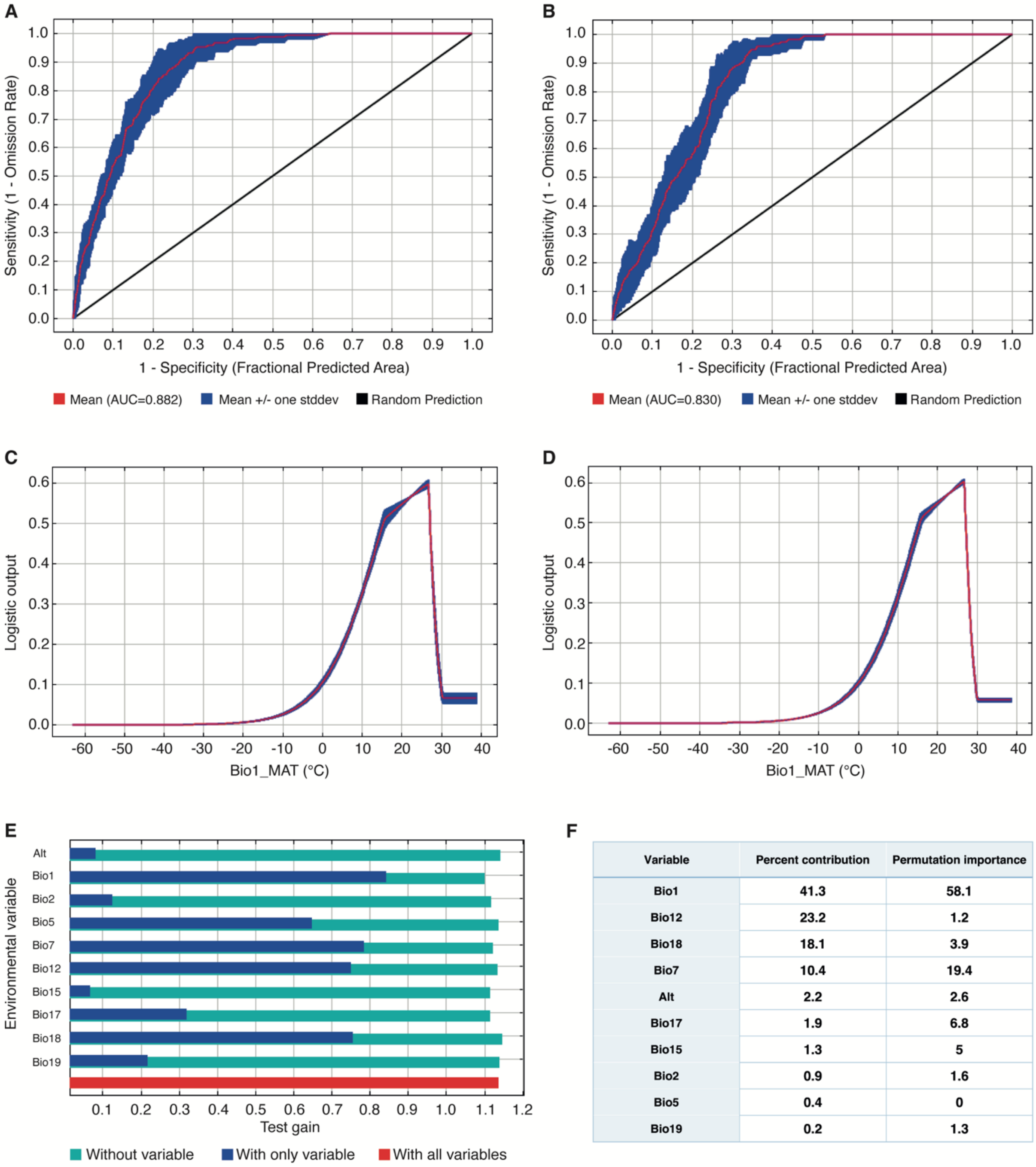

Variable	Percent contribution	Permutation importance
Bio1	41.3	58.1
Bio12	23.2	1.2
Bio18	18.1	3.9
Bio7	10.4	19.4
Alt	2.2	2.6
Bio17	1.9	6.8
Bio15	1.3	5
Bio2	0.9	1.6
Bio5	0.4	0
Bio19	0.2	1.3

Fig. S26. SDM results by Maxent. (**A**) Receiver operating characteristic (ROC) curve of Maxent model for extant mantispids using ten environmental variables; (**B**) ROC curve of Maxent model for extinct and extant mantispids using one variable; (**C**) Response curve of extant species to Bio1 (MAT); (**D**) Response curve of extinct and extant species to Bio1 (MAT); (**E**) Jackknife test of variable importance using test gain of Maxent model for extant species using ten environmental variables; (**F**) Estimates of relative contributions of the environmental variables to the Maxent model for extant species using ten environmental variables.

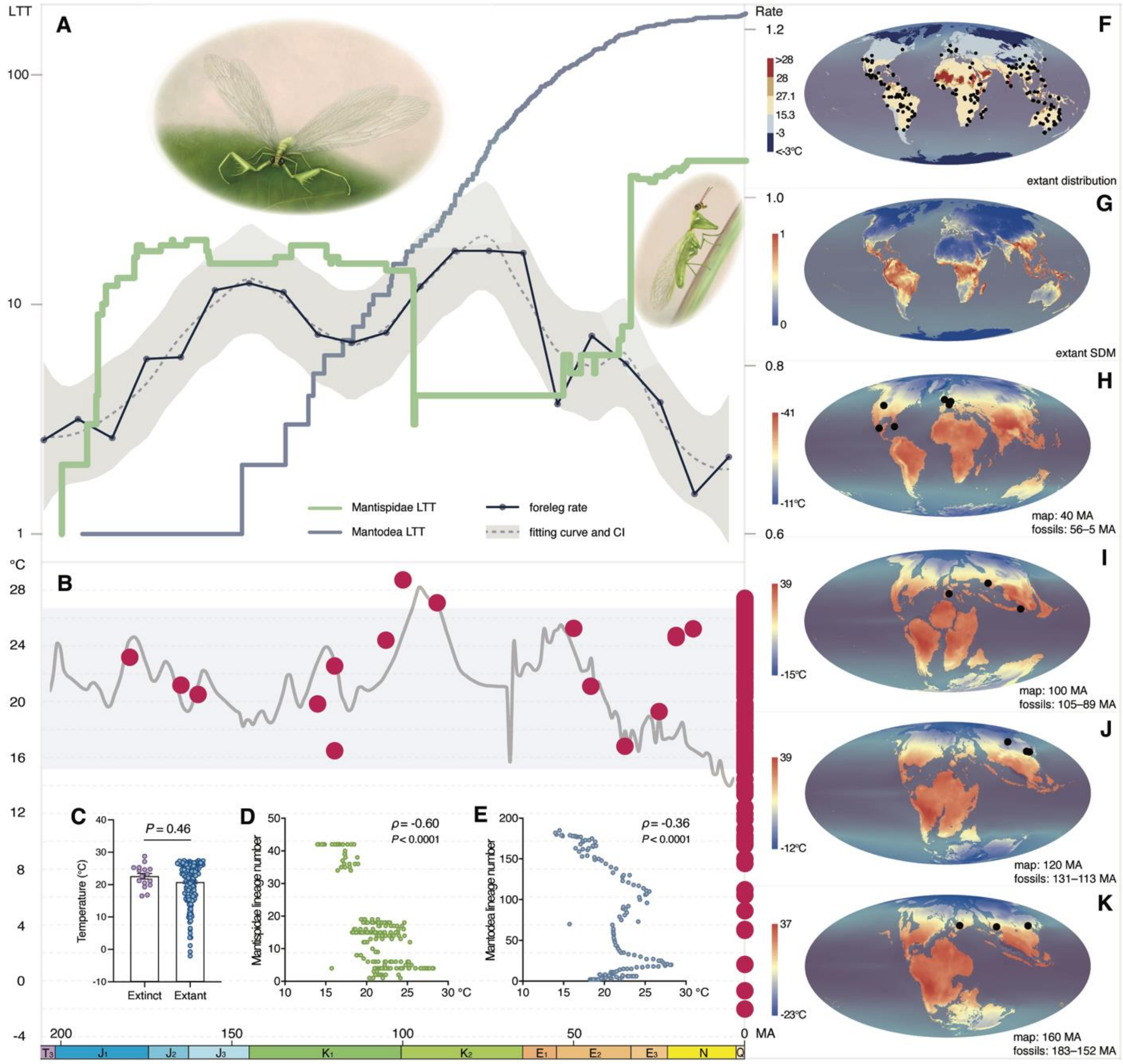

Fig. S27. Lineages through time (LTT) plots, foreleg evolutionary rates and habitat Mean Annual Temperature (MAT) in geological history and on modern earth. (**A**) Foreleg rate weighted mean per 10-million-year bin, fitting curve and 95% confidence interval plotted on LTT of Mantispidae and Mantodea in logarithmic scale. (**B**) Habitat MAT of extinct and extant mantispids in corresponding time (red dots) mapped on global average temperature variation in last 200 million years. Blue shade indicates suitable MAT range. (**C**) Extinct and extant species habitat MAT as the mean ± s.e.m. and statistical test ($n_{Extinct}$ = 16, n_{Extant} = 195). Spearman correlation between MAT and lineage number of (**D**) Mantispidae (n_{Pairs} = 205) and (**E**) Mantodea (n_{Pairs} = 148). (**F**) Distribution of extant mantispids on modern temperature zones according to their suitable level. (**G**) Extant mantispids SDM. (**H** to **K**) Distribution of extinct mantispids on paleogeography mapped with MAT of corresponding time. Paleocoordinates, paleogeography, paleo- and modern temperatures derived from refs[21,27,36,49], Fossilworks and WorldClim. Reconstructed artwork credit Yuening Jian.

Supplementary Table

Table S1. Marginal likelihood (in natural log units) for clock and rate variation model combinations estimated using stepping-stone sampling.

Clock model	Rate Model	Marginal likelihood (ln)
IGR	gamma distribution	-1158.79
IGR	lognormal distribution	-1159.13
TK02	gamma distribution	-1159.99
TK02	lognormal distribution	-1159.57

Table S2. Summary statistics for macroevolutionary parameters from the phylogenetic analysis using a single morphological clock partition.

Parameter	Time bin	n	ESS	mean	sd	median
**Net diversification**	Origin–145 Ma	32042	5820.8	0.0283	0.0133	0.0275
	145–100 Ma	32042	6644.2	0.0131	0.00996	0.0111
	100–66 Ma	32042	21093	0.00529	0.00511	0.00371
	66–0 Ma	32042	872.9	0.0174	0.00918	0.0163
**Relative extinction**	Origin–145 Ma	32042	4322.7	0.6077	0.26	0.6563
	145–100 Ma	32042	11841.9	0.3825	0.2672	0.3376
	100–66 Ma	32042	16511.4	0.4141	0.2456	0.3866
	66–0 Ma	32042	554.8	0.9834	0.0117	0.9859
**Relative fossilization**	Origin–145 Ma	32042	6570.2	0.1484	0.1583	0.0913
	145–100 Ma	32042	12365.3	0.2253	0.2146	0.1516
	100–66 Ma	32042	15390.3	0.4093	0.2447	0.3801
	66–0 Ma	32042	878.9	0.00077	0.00064	0.00058

Table S3. Characters, corresponding states and result legends of ancestral state reconstruction.

Character	State	Code	Result
**Forefemoral major spine**	Absent	0	Fig. S7A
	Basal long spine	1	
	Midway long spine	2	
	Basal extra-long spine	3	
	Midway extreme long spine	4	
**Forefemur length compared to foretibia and foretarsi together**	Significantly shorter	0	Fig. S8
	Subequal	1	
	Longer	2	
**Forefemoral appendage**	Thin setae	0	Fig. S9
	Spine-like setae	1	
	Cuticular spine	2	
**Foretibial ventral appendage**	Thin setae	0	Fig. S10
	Thick prostrate setae	1	
**Foretarsal ventral appendage**	Thin setae	0	Fig. S11
	Thick prostrate setae	1	
**Foretarsus first element apex**	Short thin setae	0	Fig. S12
	Long spine-like setae	1	
	Long cuticular setae	2	
**Foretarsal claw**	two simple	0	Fig. S13
	Two bifid	1	
	One simple	2	

Table S4. Measurements of forefemur lengths of specimens modeled in the analyses.

Genus	Forefemur length (mm)
***Clavifemora* Jepson et al., 2013**	3.3
***Longipronotum* Jepson, Khramov et Ohl, 2018**	2.7
***Archaeodrepanicus* Jepson et al., 2013**	3.8
***Lonchomantispa* Shi, Yang et Ren, 2020**	2
***Pectispina* Shi, Yang et Ren, 2020**	1.6
***Archaeosymphrasis* Shi, Yang et Ren, 2020**	2.2
***Trichoscelia* Westwood, 1852**	2.07
***Drepanicus* Blanchard in Gay, 1851**	2.7
***Nolima* Navás, 1914**	3.8
***Dicromantispa* Hoffman in Penny, 2002**	2.73
***Necyla* Navás, 1913**	3.8

Table S5. Mean value of stress on the forefemur when clamping rigid prey in FEA.

Species	von Mises stress on forefemur (MPa)
Clavifemora rotundata	0.4894
Longipronotum benmaddoxi	0.46581
Archaeodrepanicus nuddsi	0.6643
Lonchomantispa longa	3.328
Pectispina libera	3.63
Archaeosymphrasis pennyi	1.2818
Trichoscelia varia	0.24089
Drepanicus gayi	0.55216
Nolima victor	0.29429
Dicromantispa electromexicana	1.0598
Necyla minor	0.68015

Table S6. Stress on the simulated prey in mechanical experiments.

Species	Stress on simulated prey (GPa)
Clavifemora rotundata	0.26754
Longipronotum benmaddoxi	0.461
Archaeodrepanicus nuddsi	0.35637
Lonchomantispa longa	0.57498
Pectispina libera	0.14357
Archaeosymphrasis pennyi	0.28029
Trichoscelia varia	0.651
Drepanicus gayi	0.19818
Nolima victor	0.6529
Dicromantispa electromexicana	0.524925
Necyla minor	0.52486

Table S7. Force required to impale prey in mechanical experiments. * indicates the data used in the PLS analysis and Fig. 1D.

Species	Force requirement for most accessible spine (N)				Force requirement for major spine (N)			
	I	II	III	Mean*	I	II	III	Mean
Clavifemora rotundata	4.5	4.6	4.9	4.67	-	-	-	-
Longipronotum benmaddoxi	3.6	3.9	4.3	3.93	-	-	-	-
Archaeodrepanicus nuddsi	4.9	5.5	5.8	5.40	-	-	-	-
Lonchomantispa longa	0.7	0.9	0.6	0.73	1.75	1.9	2.2	1.95
Pectispina libera	1.8	2.1	2.2	2.03	2.7	2.9	2.7	2.77
Archaeosymphrasis pennyi	4	4.2	4	4.07	-	-	-	-
Trichoscelia varia	12	12.1	11.5	11.87	-	-	-	-
Drepanicus gayi	4.5	4.3	3.8	4.20	4.6	4.7	4.8	4.70
Nolima victor	1.8	2.4	1.6	1.93	3.5	3.2	3.6	3.43
Dicromantispa electromexicana	1.4	1.2	1.4	1.33	3.6	3.8	3.9	3.77
Necyla minor	3.6	3.5	4.1	3.73	4	4.3	4.1	4.13

Table S8. Effective capture range using ratio of the measured range to femur length at criterial angle.

Species	Operational capture range
Clavifemora rotundata	1.5
Longipronotum benmaddoxi	1.79
Archaeodrepanicus nuddsi	1.61
Lonchomantispa longa	1.21
Pectispina libera	0.75
Archaeosymphrasis pennyi	1.66
Trichoscelia varia	1.64
Drepanicus gayi	1.21
Nolima victor	0.97
Dicromantispa electromexicana	0.72
Necyla minor	0.73

Table S9. Selected environmental variables and descriptions used in SDM.

Variable	Description	original resolutions	Unit
**ALT**	**Altitude**	10 minutes	m
**BIO01**	**Annual Mean Temperature**	10 minutes	°C
**BIO02**	**Mean Diurnal Range (Mean of monthly (max temp - min temp))**	10 minutes	°C
**BIO05**	**Max Temperature of Warmest Month**	10 minutes	°C
**BIO07**	**Temperature Annual Range**	10 minutes	°C
**BIO12**	**Annual Precipitation**	10 minutes	mm
**BIO15**	**Precipitation Seasonality (Coefficient of Variation)**	10 minutes	-
**BIO17**	**Precipitation of Driest Quarter**	10 minutes	mm
**BIO18**	**Precipitation of Warmest Quarter**	10 minutes	mm
**BIO19**	**Precipitation of Coldest Quarter**	10 minutes	mm

Table S10. Genera of Mantispidae, annotating taxa selection in the analyses of this study.

Genus	Subfamily	Chronological constraint (Ma)	Phylogenetic analyses	Morphometrics	FEA	LTT
***Anchieta* Navás, 1909**	Symphrasinae		+	*A. eurydella*<br>*A. notha*		+
***Plega* Navás, 1928**			+	*P. sonorae*<br>*P. spinosa*		+
***Trichoscelia* Westwood, 1852**			+	*T. andina*<br>*T. gorgonensis*<br>*T. varia*	*T. varia*	+
***Archaeosymphrasis* Shi, Yang et Ren, 2020**		100.5–93.9	+	*A. pennyi*	*A. pennyi*	+
***Habrosymphrasis* Shi, Yang et Ren, 2020**		100.5–93.9	+	*H. xiai*		+
***Haplosymphrasites* Lu et al., 2020**		100.5–93.9				+
***Parasymphrasites* Lu et al., 2020**		100.5–93.9				+
***Symphrasites* Wedmann et Makarkin, 2007**		47.8–41.2	+			+
***Allomantispa* Liu et al., 2015**	Drepanicinae		+	*A. tibetana*		+
***Ditaxis* McLachlan, 1867**			+	*D. biseriata*		+
***Drepanicus* Blanchard in Gay, 1851**			+	*D. gayi*<br>*D. moulti*	*D. gayi*	+
***Gerstaeckerella* Enderlein, 1910**			+	*G. chilensis*<br>*G. irrorata*		+
Theristria			+	*T. aruntae*		+

**Gerstaecker, 1885**				*T. discolor*<br>*T. imperfecta*		
***Calomantispa* Banks, 1913**	Calomantispinae		+	*C. venusta*		+
***Nolima* Navás, 1914**			+	*N. infensa*<br>*N. victor*	*N. victor*	+
***Afromantispa* Snyman et Ohl, 2012**	Mantispinae					+
***Asperala* Lambkin, 1986**						+
***Austroclimaciella* Handschin, 1961**						+
***Austromantispa* Esben-Petersen, 1917**			+	*A. pasteuri*		+
***Buyda* Navás, 1926**						+
***Campanacella* Handschin, 1961**						+
***Campion* Navás, 1914**						+
***Cercomantispa* Handschin, 1959**						+
***Climaciella* Enderlein, 1910**			+	*C. amapaensis*<br>*C. rafaeli*		+
***Dicromantispa* Hoffman in Penny, 2002**		33.9–5.333<br><br>23.03–5.333	+<br><br>+	*D. electromexicana*<br>*D. gracilis*<br>*D. moronei*	*D. electromexicana*	+
***Entanoneura* Enderlein, 1910**						+
***Euclimacia* Enderlein, 1910**						+
***Eumantispa* Okamoto, 1910**			+	*E. fuscicolla*<br>*E. pseudoharmandi*		+

Haematomantispa **Hoffman in Penny, 2002**				+
Leptomantispa **Hoffman in Penny, 2002**				+
Madantispa **Fraser, 1952**				+
Mantispa **Illiger in Kugelann, 1798**				+
Mantispilla **Enderlein, 1910**				+
Mimetispa **Handschin, 1961**				+
Nampista **Navás, 1914**				+
Necyla **Navás, 1913**	+	*N. jucunda*<br>*N. minor*	*N. minor*	+
Paramantispa **Williner et Kormilev, 1958**				+
Paulianella **Handschin, 1960**				+
Pseudoclimaciella **Handschin, 1960**				+
Rectinerva **Handschin, 1959**				+
Spaminta **Lambkin, 1986**				+
Stenomantispa **Stitz, 1913**				+
Toolida **Lambkin, 1986**				+
Tuberonotha **Handschin, 1961**				+

***Xaviera* Lambkin, 1986**						+
***Xeromantispa* Hoffman in Penny, 2002**						+
***Zeugomantispa* Hoffman in Penny, 2002**						+
***Vectispa* Lambkin, 1986**		56–23.03	+			+
***Prosagittalata* Nel, 1988**		33.9–23.03	+			+
***Feroseta* Poinar, 2006**		23.03–5.333	+	*F. prisca*		
***Clavifemora* Jepson et al., 2013**	Mesomantispinae	168.3–166.1	+	*C. rotundata*	*C. rotundata*	+
***Karataumantispa* Jepson, 2015**		163.5–152.1	+	*K. carnaria*		+
***Longipronotum* Jepson, Khramov et Ohl, 2018**		163.5–152.1	+	*L. benmaddoxi*	*L. benmaddoxi*	+
***Ovalofemora* Jepson, Khramov et Ohl, 2018**		163.5–152.1				+
***Mesomantispa* Makarkin, 1997**		139.4–126.3				+
***Archaeodrepanicus* Jepson et al., 2013**		130.8–113	+	*A. acutus*<br>*A. nuddsi*	*A. nuddsi*	+
***Sinomesomantispa* Jepson et al., 2013**		130.8–113				+
***Doratomantispa* Poinar, 2011**	Doratomantispinae	100.5–93.9	+	*D. burmanica*<br>*D. hongi*<br>*D. pubescens*		+
***Lonchomantispa* Shi, Yang et Ren,**		100.5–93.9	+	*L. longa*	*L. longa*	+

**2020**						
***Paradoxomantispa* Lu et al., 2020**		100.5–93.9				+
***Pectispina* Shi, Yang et Ren, 2020**		100.5–93.9	+	*P. libera*	*P. libera*	+
***Liassochrysa* Ansorge et Schlüter, 1990**	*Incertae sedis*	182.7–174.1	+			+
***Promantispa* Panfilov, 1980**		163.5–152.1	+			+
***Sinuijumantispa* So et Won, 2022**		130.0–112.6				+
***Aragomantispa* Pérez-de la Fuente et Peñalver, 2019**		113–100.5				+
***Acanthomantispa* Lu et al., 2020**		100.5–93.9				+
***Dicranomantispa* Lu et al., 2020**		100.5–93.9				+
***Psilomantispa* Lu et al., 2020**		100.5–93.9				+
***Protonolima* Makarkin, 2019**		56–47.8	+	*P. mantispinoformis*		+

Table S11. List of insects with raptorial forelegs in Burmese amber.

Order	Family	Species
**Mantodea**	*Incertae sedis*	*Burmantis asiatica* Grimaldi, 2003
		Burmantis hexispinea Li et Huang, 2018
		Burmantis lebanensis Grimaldi, 2003
		Burmantis zherikhini Delclòs et al., 2016
		Jersimantis burmiticus Grimaldi, 2003
**Neuroptera**	Dipteromantispidae	*Burmodipteromantispa jiaxiaoae* Liu, Lu et Zhang, 2017
		Enigmadipteromantispa dimyi Azar, Maksoud et Huang, 2020
		Halteriomantispa grimaldii Liu, Lu et Zhang, 2016
		Kurtodipteromantispa xiai Li et al., 2020
		Kurtodipteromantispa zhuodei Li et Liu, 2020
		Mantispidipterella longissima Liu, Lu et Zhang, 2017
		Paradipteromantispa polyneura Li et al., 2020
	Mantispidae	*Acanthomantispa grandis* Lu et al., 2020
		Acanthomantispa immaculata Lu et al., 2020
		Acanthomantispa maculata Lu et al., 2020
		Archaeosymphrasis pennyi Shi, Yang et Ren, 2020
		Dicranomantispa zhouae Lu et al., 2020
		Doratomantispa arcimaculata Li et al., 2022
		Doratomantispa ares Lu et al., 2020
		Doratomantispa burmanica Poinar, 2011
		Doratomantispa gaoyuhei Li et al., 2022
		Doratomantispa hongi Shi, Yang et Ren, 2019
		Doratomantispa pouillonis Jouault, Pouillon et Nel, 2022
		Doratomantispa pubescens Lu et al., 2020

	Doratomantispa yumeiyingae Li et al., 2022
	Doratomantispa zhangwenjuni Li et al., 2022
	Doratomantispa zhuozhengmingi Li et al., 2022
	Doratomantispa zhangzhiqiae Li et al., 2022
	Habrosymphrasis xiai Shi, Yang et Ren, 2020
	Haplosymphrasites zouae Lu et al., 2020
	Lonchomantispa longa Shi, Yang et Ren, 2020
	Paradoratomantispa jiaxiaoae Lu et al., 2020
	Paradoxomantispa mahaiyingae Li et al., 2022
	Parasymphrasites electrinus Lu et al., 2020
	Pectispina libera Shi, Yang et Ren, 2020
	Psilomantispa abnormis Lu et al., 2020
Rhachiberothidae	*Acanthoberotha cuspis* Nakamine, Yamamoto et Takahashi., 2020
	Astioberotha coutreti Jouault, 2022
	Astioberotha falcipes Nakamine, Yamamoto et Takahashi, 2020
	Creagroparaberotha groehni Makarkin, 2015
	Creagroparaberotha cuneata Nakamine, Yamamoto et Takahashi, 2020
	Eorhachiberotha burmitica Engel, 2004
	Kujiberotha teruyukii Nakamine et Yamamoto, 2018
	Micromantispa cristata Shi et al., 2015
	Micromantispa galeata Nakamine, Yamamoto et Takahashi, 2020
	Micromantispa spicata Nakamine, Yamamoto et Takahashi, 2020
	Paraberothinae sp. Engel, 2004
	Paradoxoberotha chimaera Nakamine et al., 2022

	Scoloberotha necatrix Engel et Grimaldi, 2008
	Stygioberotha siculifera Nakamine, Yamamoto et Takahashi, 2020
	Uranoberotha chariessa Nakamine, Yamamoto et Takahashi, 2020

Supplementary Data list

Supplementary Data 1. Morphological matrix.

Supplementary Data 2. Foreleg 3D model of *Necyla* prepared from high resolution X-ray computed tomography scanning.

Supplementary Data 3. Foreleg 3D model of *Clavifemora rotundata*.

Supplementary Data 4. Foreleg 3D model of *Longipronotum benmaddoxi*.

Supplementary Data 5. Foreleg 3D model of *Archaeodrepanicus nuddsi*.

Supplementary Data 6. Foreleg 3D model of *Lonchomantispa longa*.

Supplementary Data 7. Foreleg 3D model of *Pectispina libera*.

Supplementary Data 8. Foreleg 3D model of *Archaeosymphrasis pennyi*.

Supplementary Data 9. Foreleg 3D model of *Trichoscelia varia*.

Supplementary Data 10. Foreleg 3D model of *Drepanicus gayi*.

Supplementary Data 11. Foreleg 3D model of *Nolima victor*.

Supplementary Data 12. Foreleg 3D model of *Dicromantispa electromexicana*.

Supplementary Data 13. Foreleg 3D model of *Necyla minor*.

Supplementary Data 14. Weighted mean of foreleg evolutionary rate across 10 My bins.

Supplementary Data 15. Source data of Fig. 1F.

Supplementary Data 16. Source data of Fig. 2G.

Supplementary Data 17. Source data of Fig. 1H.

Supplementary Data 18. Pearson correlation coefficient analysis of environmental variables, selecting criterion: $|r| < 0.8$.

References

1. Shi CF, Yang Q and Shih CK *et al*. Cretaceous mantid lacewings with specialized raptorial forelegs illuminate modification of prey capture (Insecta: Neuroptera). *Zool J Linn Soc* 2020; **190**: 1054–70.

2. Shi CF, Yang Q and Winterton SL *et al*. Stem-group fossils of Symphrasinae shed light on early evolution of Mantispidae (Insecta, Neuroptera). *Pap Palaeontol* 2020; **6**: 143–54.

3. Engel MS, Winterton SL and Breitkreuz LC. Phylogeny and evolution of Neuropterida: where have wings of lace taken us? *Annu Rev Entomol* 2018; **63**: 531–51.

4. Aspöck U and Mansell MW. A revision of the family Rhachiberothidae Tjeder, 1959, stat. n. (Neuroptera). *Syst Entomol* 1994; **19**: 181–206.

5. Macleod EG and Adams PA. A review of the taxonomy and morphology of the Berothidae, with the description of a new subfamily from Chile (Neuroptera). *Psyche* 1967; **74**: 237–65.

6. Makarkin VN, Yang Q and Ren D. Two new species of Sinosmylites Hong (Neuroptera, Berothidae) from the Middle Jurassic of China, with notes on Mesoberothidae. *ZooKeys* 2011; **130**: 199–215.

7. Nel A, Perrichot V and Azar D *et al*. New Rhachiberothidae (Insecta: Neuroptera) in Early Cretaceous and Early Eocene ambers from France and Lebanon. *Neues Jahrb Geol Palaontol Abh* 2005; **235**: 51–85.

8. Makarkin VN. New lacewings (Neuroptera) from the Late Cretaceous of Asia. In: Akimov IA (ed.). *News of Faunistics and Systematics*. Kiev: Naukova Dumka, 1990, 63–8.

9. Winterton SL, Lemmon AR and Gillung JP et al. Evolution of lacewings and allied orders using anchored phylogenomics (Neuroptera, Megaloptera, Raphidioptera). *Syst Entomol* 2017; **43**: 330–54.

10. Ardila-Camacho A, Martins CC and Aspöck U *et al*. Comparative morphology of extant raptorial Mantispoidea (Neuroptera: Mantispidae, Rhachiberothidae) suggests a non-monophyletic Mantispidae and a single origin of the raptorial condition within the superfamily. *Zootaxa* 2021; **4992**: 1–89.

11. Willmann R. The phylogenetic position of the Rhachiberothinae and the basal sister-group relationships within the Mantispidae (Neuroptera). *Syst Entomol* 1990; **15**: 253–265.

12. Haug C, Perez-De La Fuente R, Baranov V, Haug GT, Kiesmueller C, Zippel A, Hörnig MK and Haug JT. The first fossil record of a mantis lacewing pupa, and a review of pupae in Mantispidae and their evolutionary significance. *Riv Ital Paleontol S* 2023; **129**: 185–

205.
13. Lu XM, Wang B and Zhang WW et al. Cretaceous diversity and disparity in a lacewing lineage of predators (Neuroptera: Mantispidae). *Proc R Soc B* 2020; **287**: 20200629.
14. Oswald JD. Neuropterida species of the world. Version 4.0. http://lacewing.tamu.edu (2021).
15. Ronquist F, Teslenko M and van der Mark P *et al*. MrBayes 3.2: efficient Bayesian phylogenetic inference and model choice across a large model space. *Syst Biol* 2012; **61**: 539–42.
16. Heath TA, Huelsenbeck JP and Stadler T. The fossilized birth-death process for coherent calibration of divergence-time estimates. *Proc Natl Acad Sci USA* 2014; **111**: 2957–66.
17. Stadler T, Sampling-through-time in birth-death trees. *J Theor Biol* 2010; **267**: 396–404.
18. Ansorge J and Schlüter T. The earliest chrysopid: *Liassochrysa stigmatica* n.g., n. sp. from the Lower Jurassic of Dobbertin, Germany. *Neur Int* 1990; **6**: 87–93.
19. Wedmann S and Makarkin VN. A new genus of Mantispidae (Insecta: Neuroptera) from the Eocene of Germany, with a review of the fossil record and palaeobiogeography of the family. *Zool J Linn Soc* 2007; **149**: 701–16.
20. Drummond AJ, Ho SY and Phillips MJ *et al*. Relaxed phylogenetics and dating with confidence. *PLoS Biol* 2006; **4**: e88.
21. Thorne J and Kishino H. Divergence time and evolutionary rate estimation with multilocus data. *Syst Biol* 2002; **51**: 689–702.
22. Winterton SL, Lemmon AR and Gillung JP et al. Evolution of lacewings and allied orders using anchored phylogenomics (Neuroptera, Megaloptera, Raphidioptera). *Syst Entomol* 2017; **43**: 330–54.
23. Heritage S, MBASR: Workflow-simplified ancestral state reconstruction of discrete traits with MrBayes in the R environment. bioRxiv https://doi.org/10.1101/2021.01.10.426107 (2021).
24. Pérez-de la Fuente R and Peñalver E. A mantidfly in Cretaceous Spanish amber provides insights into the evolution of integumentary specialisations on the raptorial foreleg. *Sci Rep* 2019; **9**: 13248.
25. Simões TR and Pierce SE. Sustained high rates of morphological evolution during the rise of tetrapods. *Nat Ecol Evol* 2021; **5**: 1403–14.
26. Baker J, Meade A and Pagel M *et al*. Positive phenotypic selection inferred from phylogenies. *Biol J Linn Soc* 2016; **118**: 95–115.
27. Rambaut A, Suchard MA and Xie D *et al*. Tracer v1.7. http://beast.Bio.Ed.Ac.Uk/tracer

(2018).

28. Baranov V, Pérez-de la Fuente R and Engel MS et al. The first adult mantis lacewing from Baltic amber, with an evaluation of the post-Cretaceous loss of morphological diversity of raptorial appendages in Mantispidae. *Foss Rec* 2022; **25**: 11–24.

29. Svenson GJ and Whiting MF, Reconstructing the origins of praying mantises (Dictyoptera, Mantodea): the roles of Gondwanan vicariance and morphological convergence. *Cladistics* 2009; **25**: 468–514.

30. Legendre F, Nel A and Svenson GJ et al. Phylogeny of Dictyoptera: dating the origin of cockroaches, praying mantises and termites with molecular data and controlled fossil evidence. *PLoS One* 2015; **10**: e0130127.

31. Pybus OG and Harvey PH. Testing macro–evolutionary models using incomplete molecular phylogenies. *Proc R Soc B* 2000; **267**: 2267–72.

32. Revell LJ, phytools: an R package for phylogenetic comparative biology (and other things). *Methods Ecol Evol* 2012; **2**: 217–23.

33. Labandeira CC and Eble GJ. The fossil record of insect diversity and disparity. In: Anderson J, Thackeray F, Van Wyk B and De Wit M (eds.). *Gondwana Alive: Biodiversity and the Evolving Terrestrial Biosphere*. Johannesburg: Witwatersrand Univ. Press, 2000, 3–54.

34. Misof B, Liu S and Meusemann K et al. Phylogenomics resolves the timing and pattern of insect evolution. *Science* 2014; **346**: 763–7.

35. Grimaldi DA and Engel MS. *Evolution of the Insects*. Cambridge: Cambridge University Press, 2005.

36. Adams DC and Otárola-Castillo E. geomorph: an R package for the collection and analysis of geometric morphometric shape data. *Methods Ecol Evol* 2013; **4**: 393–9.

37. Adams DC and Collyer ML. Multivariate phylogenetic comparative methods: evaluations, comparisons, and recommendations. *Syst Biol* 2018; **67**: 14–31.

38. Kuhl FP and Giardina CR. Elliptic Fourier features of a closed contour. *Computer Graph Image Process* 1982; **18**: 236–58.

39. Iwata H and Ukai Y. SHAPE: a computer program package for quantitative evaluation of biological shapes based on elliptic Fourier descriptors. *J Hered* 2002; **93**: 384–5.

40. Sidlauskas B. Continuous and arrested morphological diversification in sister clades of characiform fishes: a phylomorphospace approach. *Evolution* 2008; **62**: 3135–56.

41. Slice DE. Geometric morphometrics. *Annu Rev Anthropol* 2007; **36**: 261–81.

42. Gower JC. Generalized Procrustes analysis. *Psychometrika* 1975; **40**: 33–51.

43. Rohlf FJ and Slice DE. Extensions of the Procrustes method for the optimal

superimposition of landmarks. *Syst Zool* 1990; **39**: 40–59.

44. Cambell NA and Atchley WR, The geometry of canonical variate analysis. *Syst Zool* 1981; **30**: 268–80.
45. Schlager S. Morpho and Rvcg – Shape Analysis in R: R-Packages for Geometric Morphometrics, Shape Analysis and Surface Manipulations. In: Zheng GY, Li S and Székely G (eds.). *Statistical Shape and Deformation Analysis*. New York: Academic Press, 2017, 217–56.
46. Büsse S, Bäumler F and Gorb SN. Functional morphology of the raptorial forelegs in Mantispa styriaca (Insecta: Neuroptera). *Zoomorphology* 2021; 140: 231–41
47. Hertz H. Ueber die Berührung fester elastischer Körper. *J reine und angewondte Mathematik* 1882; **92**: 156–71.
48. Redborg KE. Biology of the Mantispidae. *Annu Rev Entomol* 1998; **43**: 175–94.
49. Loxton RG and Nicholls I. The functional morphology of the praying mantis forelimb (Dictyoptera: Mantodea). *Zool J Linn Soc* 1979; **66**: 185–203.
50. Daza JD, Stanley EL and Wagner P *et al*. Mid-Cretaceous amber fossils illuminate the past diversity of tropical lizards. *Sci Adv* 2016; **2**: e1501080.
51. Daza JD, Bauer AM and Stanley EL *et al*. An enigmatic miniaturized and attenuate whole lizard from the mid-Cretaceous amber of Myanmar. *Breviora* 2018; **563**: 1–18.
52. Xing LD, Caldwell MW and Chen R *et al*. A mid-Cretaceous embryonic-to-neonate snake in amber from Myanmar. *Sci Adv* 2018; **4**: eaat5042.
53. Bell GP. Birds and Mammals on an Insect Diet: A Primer on Diet Composition Analysis. In Morrison ML, Ralph CJ, Verner J and Jehl Jr JR (eds.). *Avian Foraging: Theory, Methodology, and Applications*. Lawrence: Cooper Ornithological Society, 1990, 416–22.
54. Caldironi HA and Manes ME. Proximate composition, fatty acids and cholesterol content of meat cuts from tegu lizard *Tupinambis merianae J Food Compos Anal* 2006; **19**: 711–4.
55. Lorenzo JM, Munekata PE and Barba FJ *et al*. *More than Beef, Pork and Chicken–The Production, Processing, and Quality Traits of Other Sources of Meat for Human Diet.* Berlin: Springer, 2019.
56. Li HY, Zhuo D and Cao L *et al*. New Cretaceous fossil mantispids highlight the palaeodiversity of the extinct subfamily Doratomantispinae (Neuroptera: Mantispidae). *Org Divers Evol* 2022; **22**: 681–730.
57. Wainwright PC, Functional versus morphological diversity in macroevolution. *Annu Rev Ecol Evol Syst* 2007; **38**: 381–401.

58. Fortin MJ. Effects of sampling unit resolution on the estimation of spatial autocorrelation. *Ecoscience* 1999; **6**: 636–41.

59. Fick SE and Hijmans RJ. WorldClim 2: new 1km spatial resolution climate surfaces for global land areas. *Int J Climatol* 2017; **37**: 4302–15.

60. Scotese CR, Song HJ, and Mills BJ *et al*. Phanerozoic paleotemperatures: the earth's changing climate during the last 540 million years. *Earth-Sci Rev* 2021; **215**: 103503.

61. Scotese CR, Global mean surface temperatures for 100 Phanerozoic time intervals. https://doi.org/10.5281/zenodo.5718392 (2022).

62. Scotese CR, An atlas of Phanerozoic paleogeographic maps: the seas come in and the seas go out. *Annu Rev Earth Planet Sci* 2021; **49**: 679–728.

63. Li X, Hu YY and Guo JQ *et al*. A high-resolution climate simulation dataset for the past 540 million years. *Sci Data* 2022; **9**: 1–10.

64. Muscarella R, Galante PJ and Soley-Guardia M *et al*. ENMeval: an R package for conducting spatially independent evaluations and estimating optimal model complexity for Maxent ecological niche models. *Methods Ecol Evol* 2014; **5**: 1198–205.

65. Phillips SJ, Anderson RP and Schapire RE. Maximum entropy modeling of species geographic distributions. *Ecol Modell* 2006; **190**: 231–59.

66. Phillips SJ, Dudík M and Schapire RE. Maxent software for modeling species niches and distributions. Version 3.4.1. http://biodiversityinformatics.amnh.org/open_source/maxent/ (2020).
