# Supplementary material for: The associated evolution of raptorial foreleg and mantispid diversification during 200 million years
Source: Natl Sci Rev. 2023 Nov 2;10(12):nwad278. doi: 10.1093/nsr/nwad278 (PMC10686013; doi:10.1093/nsr/nwad278)
Supplement: nwad278_Supplemental_Files [file nwad278_supplemental_files.zip › Supplementary Data 18.docx]

| Variables | ALT | BIO1 | BIO2 | BIO3 | BIO4 | BIO5 | BIO6 | BIO7 | BIO8 | BIO9 | BIO10 | BIO11 | BIO12 | BIO13 | BIO14 | BIO15 | BIO16 | BIO17 | BIO18 | BIO19 |
| --- | --- | --- | --- | --- | --- | --- | --- | --- | --- | --- | --- | --- | --- | --- | --- | --- | --- | --- | --- | --- |
| **ALT** | 1 | -.603** | .213** | -.013 | .083 | -.648** | -.477** | .142* | -.558** | -.488** | -.734** | -.455** | -.170* | -.174* | -.128 | .076 | -.182* | -.124 | -.042 | -.183** |
| **BIO1** | -.603** | 1 | -.131 | .569** | -.647** | .738** | .917** | -.575** | .830** | .896** | .850** | .952** | .364** | .436** | .103 | .198** | .432** | .109 | .193** | .222** |
| **BIO2** | .213** | -.131 | 1 | -.133 | .293** | .308** | -.375** | .597** | -.055 | -.097 | .026 | -.209** | -.571** | -.514** | -.399** | .242** | -.510** | -.421** | -.435** | -.417** |
| BIO3 | -.013 | .569** | -.133 | 1 | -.906** | .033 | .758** | -.818** | .292** | .693** | .119 | .759** | .489** | .385** | .347** | -.064 | .384** | .360** | .142* | .454** |
| BIO4 | .083 | -.647** | .293** | -.906** | 1 | -.024 | -.858** | .934** | -.325** | -.751** | -.152* | -.848** | -.518** | -.480** | -.260** | -.009 | -.481** | -.279** | -.219** | -.382** |
| **BIO5** | -.648** | .738** | .308** | .033 | -.024 | 1 | .438** | .109 | .737** | .579** | .947** | .525** | -.066 | .047 | -.150* | .257** | .043 | -.159* | -.112 | -.071 |
| BIO6 | -.477** | .917** | -.375** | .758** | -.858** | .438** | 1 | -.845** | .648** | .909** | .604** | .981** | .536** | .535** | .268** | .036 | .532** | .283** | .252** | .401** |
| **BIO7** | .142* | -.575** | .597** | -.818** | .934** | .109 | -.845** | 1 | -.278** | -.661** | -.105 | -.773** | -.632** | -.564** | -.385** | .113 | -.563** | -.408** | -.346** | -.486** |
| BIO8 | -.558** | .830** | -.055 | .292** | -.325** | .737** | .648** | -.278** | 1 | .554** | .842** | .700** | .224** | .347** | -.005 | .306** | .338** | -.009 | .255** | .035 |
| BIO9 | -.488** | .896** | -.097 | .693** | -.751** | .579** | .909** | -.661** | .554** | 1 | .654** | .927** | .377** | .377** | .176* | .035 | .377** | .187** | .081 | .333** |
| BIO10 | -.734** | .850** | .026 | .119 | -.152* | .947** | .604** | -.105 | .842** | .654** | 1 | .652** | .117 | .226** | -.032 | .225** | .221** | -.038 | .076 | .042 |
| BIO11 | -.455** | .952** | -.209** | .759** | -.848** | .525** | .981** | -.773** | .700** | .927** | .652** | 1 | .460** | .489** | .181* | .129 | .487** | .193** | .212** | .314** |
| **BIO12** | -.170* | .364** | -.571** | .489** | -.518** | -.066 | .536** | -.632** | .224** | .377** | .117 | .460** | 1 | .868** | .734** | -.282** | .897** | .760** | .691** | .746** |
| BIO13 | -.174* | .436** | -.514** | .385** | -.480** | .047 | .535** | -.564** | .347** | .377** | .226** | .489** | .868** | 1 | .378** | .116 | .989** | .403** | .731** | .531** |
| BIO14 | -.128 | .103 | -.399** | .347** | -.260** | -.150* | .268** | -.385** | -.005 | .176* | -.032 | .181* | .734** | .378** | 1 | -.630** | .421** | .994** | .378** | .762** |
| **BIO15** | .076 | .198** | .242** | -.064 | -.009 | .257** | .036 | .113 | .306** | .035 | .225** | .129 | -.282** | .116 | -.630** | 1 | .073 | -.637** | -.012 | -.444** |
| BIO16 | -.182* | .432** | -.510** | .384** | -.481** | .043 | .532** | -.563** | .338** | .377** | .221** | .487** | .897** | .989** | .421** | .073 | 1 | .444** | .747** | .545** |
| **BIO17** | -.124 | .109 | -.421** | .360** | -.279** | -.159* | .283** | -.408** | -.009 | .187** | -.038 | .193** | .760** | .403** | .994** | -.637** | .444** | 1 | .389** | .780** |
| **BIO18** | -.042 | .193** | -.435** | .142* | -.219** | -.112 | .252** | -.346** | .255** | .081 | .076 | .212** | .691** | .731** | .378** | -.012 | .747** | .389** | 1 | .204** |
| **BIO19** | -.183** | .222** | -.417** | .454** | -.382** | -.071 | .401** | -.486** | .035 | .333** | .042 | .314** | .746** | .531** | .762** | -.444** | .545** | .780** | .204** | 1 |

^*^ *P* < 0.05, ^**^ *P* < 0.01.
